# Ion-tunable antiambipolarity in mixed ion–electron conducting polymers enables biorealistic organic electrochemical neurons

**DOI:** 10.1038/s41563-022-01450-8

**Published:** 2023-01-12

**Authors:** Padinhare Cholakkal Harikesh, Chi-Yuan Yang, Han-Yan Wu, Silan Zhang, Mary J. Donahue, April S. Caravaca, Jun-Da Huang, Peder S. Olofsson, Magnus Berggren, Deyu Tu, Simone Fabiano

**Affiliations:** 1 Laboratory of Organic Electronics, Department of Science and Technology, Linköping University, Norrköping, Sweden; 2 Wallenberg Wood Science Center, Linköping University, Norrköping, Sweden; 3 Laboratory of Immunobiology, Division of Cardiovascular Medicine, Department of Medicine, Solna, Karolinska Institutet, Stockholm, Sweden; 4 n-Ink AB, Norrköping, Sweden

**Keywords:** Electronic devices, Electrical and electronic engineering, Electronic materials

## Abstract

Biointegrated neuromorphic hardware holds promise for new protocols to record/regulate signalling in biological systems. Making such artificial neural circuits successful requires minimal device/circuit complexity and ion-based operating mechanisms akin to those found in biology. Artificial spiking neurons, based on silicon-based complementary metal-oxide semiconductors or negative differential resistance device circuits, can emulate several neural features but are complicated to fabricate, not biocompatible and lack ion-/chemical-based modulation features. Here we report a biorealistic conductance-based organic electrochemical neuron (c-OECN) using a mixed ion–electron conducting ladder-type polymer with stable ion-tunable antiambipolarity. The latter is used to emulate the activation/inactivation of sodium channels and delayed activation of potassium channels of biological neurons. These c-OECNs can spike at bioplausible frequencies nearing 100 Hz, emulate most critical biological neural features, demonstrate stochastic spiking and enable neurotransmitter-/amino acid-/ion-based spiking modulation, which is then used to stimulate biological nerves in vivo. These combined features are impossible to achieve using previous technologies.

## Abstract (web summary)

Silicon-based complementary metal-oxide semiconductors or negative differential resistance device circuits can emulate neural features, yet are complicated to fabricate and not biocompatible. Here, the authors report an ion-modulated antiambipolarity in mixed ion–electron conducting polymers demonstrating capability of sensing, spiking, emulating the most critical biological neural features, and stimulating biological nerves in vivo.

## Main

Neurons communicate and process information using action potentials or spikes in membrane potentials^1^. This spike generation and the properties of the neuron are defined by voltage-, ion- and neurotransmitter-dependent conductance of various ion channels in the cell membrane. On receiving an electrical input, the cumulative effect of ionic currents through these channels—primarily the sodium and potassium channels—perturbs the membrane voltage from its resting value, resulting in an action potential. Thus, to create a realistic electrical circuit analogue to the biological neuron, one should emulate the conductance of the sodium channel, which shows a fast activation and inactivation, and the potassium channel, which activates after a certain delay. Simple leaky integrate-and-fire model neurons, based on silicon^2^ or organic semiconductors^3,4^, do not incorporate such complex ion channel dynamics and hence can emulate only limited neural features. More biorealistic conductance-based neuron models^4,5^ can be realized using silicon-based complementary metal-oxide semiconductor circuits^2,5^, Mott-memristor-based negative differential resistance (NDR) devices^6,7^ and antiambipolar p–n heterojunction^8^ transistors. However, these artificial neurons are restricted to pure electrical modulation of neural features, do not explore the ion-/neurotransmitter-based modulation mechanisms of real biological neurons and generally operate at time scales and spike voltage swings drastically different from those found in biology. As a result, these circuits do not facilitate integration with biology or direct sensing/processing of biological, chemical or physical stimuli at the neuron and require coupling of additional sensing elements to act as event-based neuromorphic sensing/processing elements^9^. Organic electrochemical transistors (OECTs) based on mixed ion–electron conducting polymers are attractive in this context due to their sensing capabilities (biological, chemical and physical signals), biocompatibility, biorealistic switching speeds, low operating voltages and coupled ionic–electronic transport properties which are amenable to modulation by external (ion/molecule) dopants^10,11^. Recently, we observed that rigid conjugated polymers such as the ladder-type poly(benzimidazobenzophenanthroline) (BBL) exhibit a reduction in the electrical conductivity on high electrochemical doping (>0.8 electrons per monomer) due to the formation of multiply charged species with reduced mobility^12^.

In this article, we utilize this ion-tunable antiambipolarity to emulate the activation and inactivation of sodium channels in biological neurons and hence realize a conductance-based organic electrochemical neuron (c-OECN). This c-OECN can spike at bioplausible frequencies (100 Hz), replicate most neural features and exhibit stochastic response in the presence of noise. Since the antiambipolar element is responsive to ions and biomolecules, we demonstrate that this neuron can be operated as an event-based sensor transducing such biochemical signals to actuate/stimulate the vagus nerve of a mouse, showing the potential for c-OECN-based closed-loop regulation of physiology.

## Antiambipolarity in BBL and its modulation

**Fig. 1:**
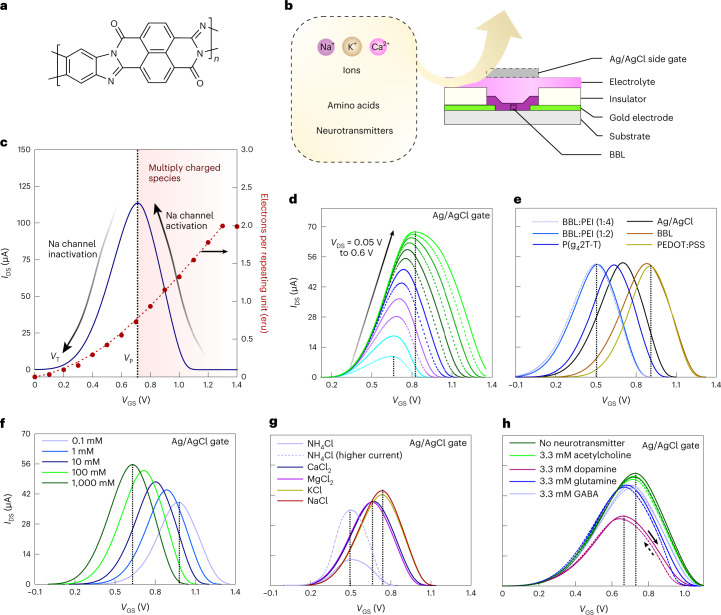
Antiambipolarity in BBL and its modulation. **a**, Structure of BBL. **b**, Structure of an OECT device. **c**, The antiambipolar behaviour in BBL resembles the activation and inactivation of sodium channels in a neuron. **d**–**h**, Modulation of the antiambipolar behaviour by electrical and chemical means, showing the effects of *V*_DS_ dependence (**d**), different gate electrodes (**e**), ion concentration (**f**), ion type (**g**) and different amino acids/neurotransmitters (**h**). The OECT used in the comparison has a *W*/*L* = 40 µm/6 µm and 20-nm-thick BBL except for the higher-current NH_4_Cl device, which uses a wider channel (*W/L* = 400 µm/6 µm). A concentration of 100 mM is used for comparing various types of ions. Neurotransmitter and amino acid studies are carried out in 100 mM NaCl. The vertical dashed lines in **d**–**h** denote the gate voltage corresponding to the peak drain current (*V*_P_). The solid and dashed lines in **h** denote the forward and reverse scans. Source data

When used as the channel material in OECTs, BBL exhibits a unique, stable and reversible Gaussian-shaped transfer curve (or antiambipolar behaviour) which is similar to voltage-controlled NDR but here instead realized in a three-terminal configuration (Fig. 1a–c and Supplementary Figs. 1–4). When implemented in a circuit, the two sides of this Gaussian current evolution can be analogous to the activated and inactivated states of the voltage-gated sodium channel in the Hodgkin–Huxley neuron model (HH model; Supplementary Note 1)^13^. Although such antiambipolar behaviour can also be observed in other n- and p-type polymers (Supplementary Figs. 5–7), only BBL with suitable electron affinity (4.15 eV (ref. 14)) and a rigid ladder-like structure composed of double-strand chains linked by condensed *π*-conjugated units can sustain such high doping levels without any conformational disorder^15^, enabling reversibility.

The OECT configuration provides improved control over the antiambipolar response compared with a conventional two-terminal NDR device. For example, applying a higher drain voltage (*V*_DS_) increases the peak current, causes a shift in the voltage of the peak current (*V*_P_) and results in a Gaussian distribution of a greater full-width at half maximum (Fig. 1d) due to variable doping levels at the drain and source electrodes. The *V*_P_ and threshold voltage (*V*_T_) can also be shifted and controlled by using gate electrodes of appropriate work function (Fig. 1e) and by tuning the concentration of ions in the electrolyte (Fig. 1f). In addition, for a given concentration (100 mM), different ions shift the transfer curve by varying degrees, with Ca^2+^, Mg^2+^ and NH_4_^+^ showing lower *V*_P_ than Na^+^ and K^+^ (Fig. 1g). Interestingly, various ammonium-based organic cations also lead to unique Gaussian behaviours (Supplementary Fig. 8), unlocking the possibility of chemical-specific responses. Hence it is possible to tune the antiambipolar response using different neurotransmitters and amino acids such as acetylcholine, dopamine, γ-butyric acid (GABA) and glutamine with different configurations of amine groups (Fig. 1h). Adding 3.3 mM acetylcholine to the 100 mM NaCl electrolyte of the OECT does not shift the *V*_P_, whereas the same concentration of dopamine, GABA or glutamine causes changes in *V*_P_ and the channel’s conductance. We attributed this effect to hydrogen-bonding interactions of these molecules with BBL (Supplementary Note 1 and Supplementary Figs. 9–14).

## Conductance-based organic electrochemical neuron

**Fig. 2:**
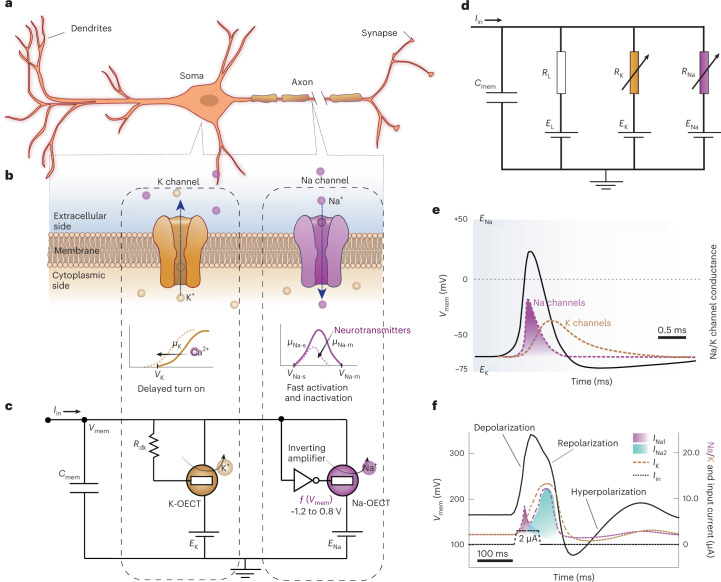
Conductance-based organic electrochemical neuron. **a**–**c**, Analogy between biological neurons (**a**) showing Na^+^ and K^+^ channels (**b**) and the c-OECN circuit with Na^+^- and K^+^-based OECTs (**c**) and their modulation with Ca^2+^ and neurotransmitters. *C*_mem_ is optional in the circuit and can be embedded in the intrinsic capacitance of the OECT. **d**, The Hodgkin–Huxley circuit of the neuron. **e**,**f**, Comparison of the squid axon action potential studied by Hodgkin and Huxley^13^ (**e**) and the c-OECN action potential (**f**). *C*_mem_ is the membrane capacitance, *V*_mem_ the membrane voltage, *I*_in_ the input current and *R*_dk_ the resistance which induces delay. *E*_L_ (*R*_L_), *E*_K_ (*R*_K_) and *E*_Na_ (*R*_Na_) represent the leakage, potassium and sodium batteries (resistances) in the HH model. *V*_K_ and *μ*_K_ are the threshold voltage and mobility of the K-OECT. *V*_Na-s_ (*μ*_Na-s_) and *V*_Na-m_ (*μ*_Na-m_) are the threshold voltage (mobility) on the low- and high-voltage sides of the antiambipolar transfer curve of Na-OECT representing the single and multiply charged species. *I*_Na_ and *I*_K_ are the sodium and potassium currents. Part **e**: copyright 1952 Wiley. Adapted with permission from A.L. Hodgkin, A.F. Huxley, A quantitative description of membrane current and its application to conduction and excitation in nerve, *Journal of Physiology*, John Wiley and Sons. Source data

The c-OECN circuit described here uses two OECTs—one Na^+^-based (Na-OECT) and another K^+^-based (K-OECT)—coupled to two voltage sources *E*_Na_ (500 mV) and *E*_K_ (∼12 to −72 mV) like the two ion channels and batteries in the HH model^13,16^ (Fig. 2a–d). *E*_Na_ is applied to the drain of Na-OECT and *E*_K_ to the source of K-OECT. The switching speed of these OECTs is in the range of 0.5–1 ms (ref. 17) (Supplementary Figs. 15 and 16), which is comparable to the time scales of sodium and potassium channel activation in biological neurons. The K-OECT channel has a thicker BBL film (50 nm compared with 20 nm for the Na-OECT) to allow higher currents through the potassium channel.

A current *I*_in_ (in the range 0.5–6 μA) injected into this circuit is integrated by the capacitance *C*_mem_ causing the voltage *V*_mem_ to increase from its resting value of ∼175 mV. This simultaneously sweeps the voltage on the gate of Na-OECT from ∼1.2 V to ∼0.8 V using an n-type metal-oxide semiconductor-based inverting amplifier (Supplementary Fig. 17) to traverse the antiambipolar transfer curve through *V*_P_, producing a spike in current *I*_Na1_. This current spike charges the capacitor even further, resulting in a rapid increase of *V*_mem_ to ∼0.34 V (depolarization). The K-OECT, which turns on after a delay enabled by its intrinsic gate capacitance and resistance *R*_dk_, reaches its peak current after Na-OECT has crossed its maximum at *V*_P_. The capacitor is thus discharged through K-OECT, causing the *V*_mem_ to drop (repolarization) and traversing the Gaussian response to its initial state back through *V*_P_, causing a secondary spike *I*_Na2_. The secondary spike in Na-OECT (not present in the squid axon action potential; Fig. 2e,f) is unnecessary for spike generation but is unavoidable, hence is entirely discharged by the K-OECT (due to the higher conductivity of K-OECT) not to cause any further voltage increase. Since the current of K-OECT is higher and persists longer, the voltage is brought below the resting value of 175 mV for a brief period (hyperpolarization). All these processes repeat cyclically if the input current remains constant, resulting in continuous action potential generation (tonic spiking). The c-OECN action potential thus shows typical features of a biological action potential, including depolarization, repolarization and hyperpolarization (Fig. 2f, Supplementary Figs. 18–25 and Supplementary Video 1). Supplementary Note 4 provides the circuit analysis of the c-OECN and the SPICE simulation and compares the circuit equations with the HH model. Note that *V*_DS_ for Na-OECT is not constant because the voltage at the source side is *V*_mem_, which continuously fluctuates. Hence, the effective *V*_DS_ of Na-OECT is *E*_Na_ – *V*_mem_. Similarly, *V*_mem_ acts at the drain of the K-OECT, and thus its effective *V*_DS_ is *V*_mem_ – *E*_k_.

## Features of the c-OECN

**Fig. 3:**
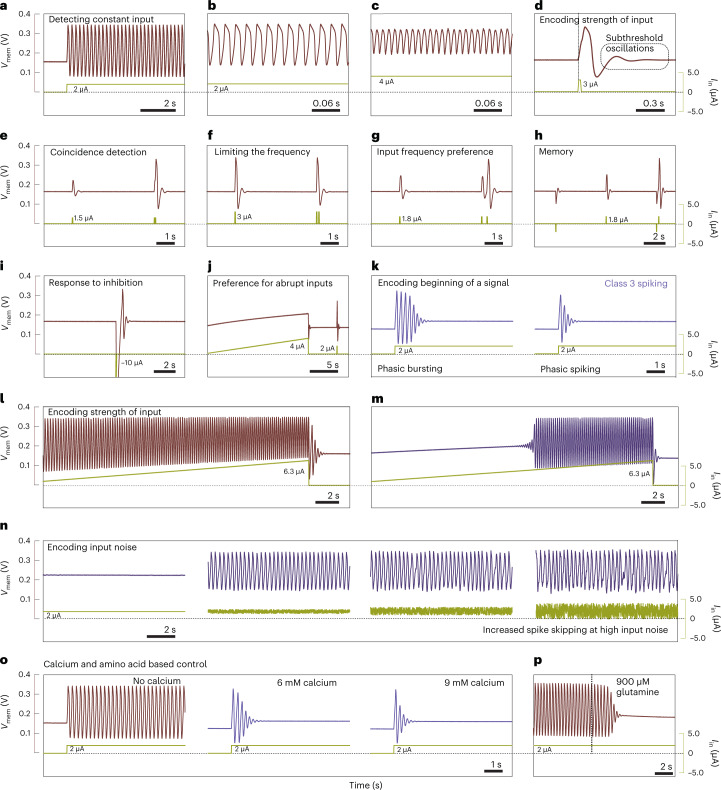
Experimental demonstration of various neural features using c-OECN and their functions in biology. **a**–**p**, Tonic spiking (**a**), higher-frequency tonic spiking by modulating *R*_dk_ and *C*_mem_ (45 Hz, **b**; 80 Hz, **c**), latency (**d**), integration (**e**), refractoriness (**f**), resonance (**g**), threshold variability (**h**), rebound spike (**i**), accommodation (**j**), class 3 spiking—phasic bursting and spiking (**k**), class 1 spiking (**l**), class 2 spiking (**m**), stochastic spiking with noisy input (**n**), calcium-based modulation from class 1 to class 3 spiking (**o**) and modulation of spiking using the amino acid glutamine (**p**). The *x* axis of all the graphs represents time. *V*_mem_ and *I*_in_ are the membrane voltage and input current. The parameters used to enable these behaviours are listed in Supplementary Table 1. Source data

The resemblance between the operation of the c-OECN and that of the biological neuron means that several neural features^17^ can be mimicked by modulating the threshold and currents of OECTs (Fig. 3 and Supplementary Table 1). In the standard configuration, the neuron exhibits tonic spiking, that is, excitability in the presence of a constant input while remaining quiescent otherwise. Tuning the capacitance *C*_mem_ and the resistance *R*_dk_ can tune the frequency of this spiking. The c-OECN spikes at a frequency of around 5 Hz with a *C*_mem_ = 1 μF and *R*_dk_ = 470 kΩ (Fig. 3a) and can be increased to reach 45 Hz or 80 Hz (Fig. 3b,c) by excluding external capacitance and then utilizing only the internal capacitance of the OECT. Furthermore, the n-type metal-oxide semiconductor-based inverting amplifier can be replaced with an OECT-based amplifier to achieve spiking at 100 Hz (Supplementary Figs. 26 and 27). Hence, biorealistic frequencies can be achieved with a neuron based on three OECTs. For ease of measurement of neural features with various pulsed inputs, we used the lower-frequency c-OECN (~5 Hz) by employing *C*_mem_ = 1 μF. The peak power (energy) consumption of the circuit spiking at 80 Hz is around 60 μW (175 nJ per spike). Supplementary Note 5 discusses strategies to lower energy consumption.

Most neural features such as tonic spiking, latency, subthreshold oscillations, integration, refractoriness, resonance, threshold variability, rebound spiking, accommodation, phasic spiking, phasic bursting, and class 1 and class 2 excitability can be demonstrated using this circuit (Fig. 3d–m). Each of these has a specific function or serves as a mathematical operator in a neuron (Supplementary Note 6). Switching from class 1 (input-strength-dependent excitability) to class 2 (spiking only at high current inputs with a high frequency) and class 3 spiking (spiking/bursting only at the beginning of input) can be enabled by simple tuning of the threshold of the K-OECT (by changing *E*_k_) to modify the relative timing of it turning on. For example, for the c-OECN demonstrated here, an *E*_k_ of −50 mV results in a class 1 spiking neuron, while causing K-OECT to turn on earlier by changing *E*_k_ to −65 mV changes the behaviour to class 2 spiking, and increasing it to a more negative value of −70 mV (−72 mV) results in phasic bursting (phasic spiking) which is class 3 behaviour. Incorporating additional channels in the circuit similar to the Ca^2+^ modulated channels in biological neurons^18^ may enable other missing features achievable by biological neurons such as tonic bursting, mixed-mode spiking, spike frequency adaptation, bistability and inhibition-induced spiking^17^.

The c-OECN can also exhibit input-noise-dependent stochasticity or spike skipping, like the biological neurons^19^. The class 2 neuron shown in Fig. 3n does not spike at an input current of 2 μA. However, when very low noise is superimposed on this input, keeping the average current the same, it starts spiking at a particular frequency—increasing the input noise results in random skipping of spikes while keeping this base frequency constant. A similar mechanism is observed in biological neurons, for example, in mammalian cold thermoreceptors^19^ where temperature-induced noise causes stochasticity in spiking and is used to extend the range of encodable stimuli. Such stochastic spiking can also enable probabilistic neural sampling and finds application in spike-based Bayesian learning and inference^20^.

## Modulation using (bio)chemical signals and biointegration

The unique feature of the c-OECN compared to other conductance-based circuit realizations of neurons is that it can be controlled using secondary ions such as Ca^2+^ and neurotransmitters as they can affect the *V*_P_ and the maximum current of the Gaussian transfer curve. In biological neurons, Ca^2+^ plays crucial roles in regulating neural activity by modifying the opening and closing of sodium and potassium channels, stimulating the release of neurotransmitters leading to synaptic plasticity, and even regulating metabolism and cell growth^21^. Inspired by this, we tried to modulate the potassium channel of the c-OECN using external Ca^2+^ ions. Incorporating Ca^2+^ ions into the electrolyte of the K-OECT shifts its threshold *V*_K_ towards lower values. This is equivalent to *V*_K_ modulation by altering *E*_K_ as described above and hence creates the same effect, that is, a shift from class 1 spiking to phasic bursting and finally phasic spiking on a slow increase of Ca^2+^ concentration (Fig. 3o). Such a transition is similar to the case in biological neurons, where the generation of phasic firing is known to be Ca^2+^ ion concentration dependent^22^. In addition to ions, biological neurons are also affected by the presence of neurotransmitters and amino acids. For example, GABA is an inhibitory neurotransmitter in the brain^23^ and inhibits the generation of action potentials by increasing chloride or potassium ion conductance and hyperpolarizing the membrane. Here we coupled GABA to the Na-OECT, and since GABA reduces the maximum current of the Na-OECT and shifts its *V*_P_ towards lower values, the sodium current spike induced depolarization, and hence spiking is instantly inhibited in the c-OECN, thus enabling neurotransmitter-induced modulation of spiking similar to biology (Supplementary Fig. 41). Similarly, the c-OECN can be controlled by the amino acid glutamine such that it stops spiking above biologically relevant concentrations of around 1 mM (ref. 24) (Fig. 3p).

**Fig. 4:**
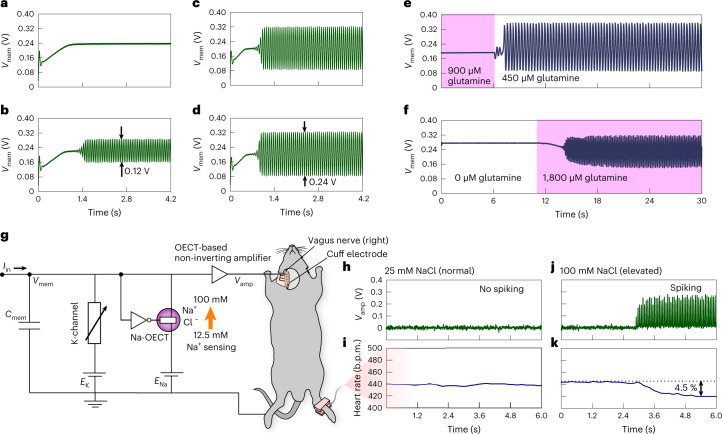
Modulation using (bio)chemical signals and biointegration. **a**–**d**, Modulation of spiking of c-OECN based on NaCl concentrations of 12.5 mM (**a**), 25 mM (**b**), 50 mM (**c**) and 100 mM (**d**). **e**,**f**, A neuron optimized to spike at low glutamine concentrations (<900 μM) (**e**) and at high glutamine concentrations (>1,800 μM) (**f**). **g**, The c-OECN circuit showing sensing of Na^+^ ions at the Na-OECT and integration with the vagus nerve using an OECT-based amplifier and cuff electrodes. **h**,**i**, The amplifier output at a low NaCl concentration of 25 mM (**h**) and the corresponding heart rate variation (**i**). **j**,**k**, The amplifier output at a high NaCl concentration of 100 mM (**j**) and the corresponding heart rate variation (**k**). The black horizontal dashed line in **k** represents the baseline heart rate. The purpose of this demonstration is to show the potential of c-OECN to sense biochemical signals and interface with nerves and does not imply that new therapeutic means are developed. Source data

To further illustrate the possibility of using c-OECNs in sensing, biointegration and nerve activation, we demonstrate event-based sensing of Na^+^ and glutamine (Fig. 4a–f) at the neuron combined with stimulation of the right cervical vagus nerve of a mouse to control its heart rate. Vagus nerve stimulation (VNS) is used as a therapeutic intervention for treating depression, controlling epileptic seizures and in clinical trials for treating chronic inflammatory diseases such as rheumatoid arthritis and inflammatory bowel disease^25,26^. Experimental animal models (commonly genetic mouse models) are often used to study the physiological effects and mechanisms of VNS and to estimate its therapeutic potential. Here we coupled the c-OECN with a mouse’s right cervical vagus nerve, which innervates the sinoatrial node where the pacemaker cells for heart rate are located, using a cuff electrode^27^ (Supplementary Fig. 43), and monitored the heart rate. We observed a 4.5% reduction in heart rate in response to an increased Na^+^ concentration sensed at the Na-OECT, consistent with electrical activation of the right cervical vagus nerve (Fig. 4g–k). As discussed previously, the NaCl concentration can substantially shift the *V*_P_ of the Gaussian transfer curve (Fig. 1f) of the Na-OECT to modulate the spiking of the c-OECN, thus enabling event-based ion sensing at the neuron without external sensors (Fig. 4a–d). Sensing Na^+^ has high clinical significance in detecting diseases such as cystic fibrosis, where the sweat Na^+^ and Cl^−^ concentrations can increase to high levels (>60 mM) compared with baseline (<30 mM)^28^. The c-OECN can be made to spike only at a high NaCl concentration (>25 mM) relevant to this condition (by choosing appropriate values of *V*_K_, *V*_Na_ and inverter threshold) and resulting in an actuation; here, for example, the VNS-based control of heart rate. The sensing capability of the c-OECN is not limited to ions. It can also be made to spike only at either a low (<900 μM) or high (>1,800 μM) glutamine concentration in the biologically relevant range and combined with stimulation of the vagus nerve. The demonstration of sensor-triggered vagus nerve activation does not imply that a new therapeutic means is developed in this study but shows the potential for future c-OECN-based closed-loop regulation of physiology. Considering the key role the vagus nerve plays in regulating homeostasis, including immune-system and metabolic control^29^, VNS triggered by event-based internal sensing has interesting potential therapeutic applications, for example, in improving the regulation of cytokine release or glucose levels to treat autoimmune or metabolic diseases. This notion may be extended to sensor-based regulation of other aspects of normal physiology and pathological conditions through peripheral nerve activation, such as VNS and voltage-triggered drug delivery.

In conclusion, we demonstrated a biorealistic OECN based on highly tunable, stable and reversible antiambipolar behaviour in BBL-based OECTs. Two OECTs modulated by Na^+^ and K^+^ ions resemble the voltage-gated ion channels in biological neurons, enabling various neural features and facile sensing and integration with the vagus nerve of a mouse. A comparison of the c-OECN with other neuron technologies is provided in Supplementary Table 2. The intrinsic capacitance of the OECTs and the biorealistic switching speeds circumvent the need for additional capacitors required in silicon-based circuits to spike at biologically plausible frequencies for interaction with real-world events and biological neurons^30^. Alternative implementations using Mott-memristors that can exhibit similar features are inherently faster than biology (ns to μs) and are associated with unfavourable temperature increases on operation, making them unsuitable for biointegration. In addition, using single-polymer material to achieve Gaussian behaviour similar to NDR averts complex fabrication strategies and helps achieve lower device dimensions compared with the heterojunction approach in 2D materials or p–n junction polymers. The ion-, amino acid- and neurotransmitter-based modulation in this c-OECN is unprecedented and may be extendable to other biomolecules that can interact with BBL. Although the sensing of molecules is done externally in the current study, it demonstrates the capability of tuning the c-OECN to respond to a specific concentration of biochemical signal by simple modulation of voltages in the circuit to enable event-based sensing. Similar stable ladder-like conjugated polymers functionalized to interact with specific biomolecules are a possible way forward to realize intelligent closed-loop event-based internal sensing and feedback neuromorphic biomedical systems and future brain–machine interfaces.

## Methods

### Materials

Poly(3,4-ethylenedioxythiophene) polystyrene sulfonate (PEDOT:PSS, Clevios PH1000) was purchased from Heraeus Holding. Naphthalenetetracarboxylic dianhydride (NDA), 1,2,4,5-tetraaminobenzene tetrahydrochloride (TABH), poly(phosphoric acid) (PPA), methanesulfonic acid (MSA), chloroform, 1,2-dichlorobenzene, ethylene glycol, (3-glycidyloxypropyl)trimethoxysilane and 4-dodecylbenzenesulfonic acid were purchased from Sigma-Aldrich. BBL (*η* = 6.3 dl g^−1^ in MSA at 30 °C, *M*_w_ = 35 kDa) was made by polycondensation of NDA and TABH in PPA at elevated temperatures^17,31^.

### Thin-film casting

BBL was dissolved in MSA at 100 °C for 12 h, followed by cooling to room temperature to obtain the BBL–MSA solution. This solution was spin-coated (1,000 r.p.m., 60 s, acceleration 1,000 r.p.m. s^−1^) on OECT substrates. The residual MSA in the films was removed by immersing them in deionized water, followed by drying in nitrogen flow. PEDOT:PSS, ethylene glycol, (3-glycidyloxypropyl)trimethoxysilane and 4-dodecylbenzenesulfonic acid were mixed in the volume ratio of 100:5:1:0.1 and sonicated for 10 min. This solution was spin-coated (2,000 r.p.m., acceleration 2,000 r.p.m. s^−1^, 60 s) on OECT substrates and annealed at 120 °C for 1 min for crosslinking PEDOT:PSS.

### OECT fabrication and testing

OECTs were fabricated according to a previous protocol^17,32^. Four-inch glass wafers were cleaned with acetone, deionized water and isopropyl alcohol and then dried using nitrogen. Electrodes (5 nm chromium and 50 nm gold) were thermally evaporated and then patterned by photolithography. A layer of parylene carbon (PaC, 1 µm) was then deposited in the presence of 3-(trimethoxysilyl)propyl methacrylate (A-174 Silane) (to increase adhesion). This forms an insulating layer to prevent unwanted capacitive effects at the electrode–electrolyte interface. After that, an antiadhesive layer of industrial surfactant (2% Micro-90) was spin-coated, and a sacrificial 2-µm-thick layer of PaC was deposited. A 5-µm-thick AZ10XT520CP positive photoresist was then spin-coated on this layer. This protects the PaC layers from the following plasma reactive ion etching (RIE) step (150 W, 500 sccm O_2_, 100 sccm CF_4_, 380 s). Another photolithographic patterning was performed to define the contact pads and the OECT channel, and the AZ developer was applied to the photoresist. A plasma RIE was carried out to remove the organic materials (photoresist and PaC), exposing the OECT channel area and the contact pads. The remaining surface remained covered with layers of PaC. This was followed by patterning the OECT channel to obtain a width/length (*W*/*L)* = 40 µm/6 µm or 80 µm/6 µm (for Na-OECTs), and 400 µm/6 µm (for K-OECTs). A 20-nm-thick (Na-OECT) or 50-nm-thick (K-OECT) film was obtained by spin-coating the BBL–MSA solution described previously. The sacrificial parylene layer was then peeled off to remove the unwanted BBL film outside the electrode area. This leaves separated pieces of semiconductor film confined to the wells, connecting the OECT source/drain electrodes. Ag/AgCl paste was drop-cast on the substrate to form a 1-μm-thick, 9 mm^2^ square gate electrode. OECTs based on PEDOT:PSS, P(g_4_2T-T) and p(g_7_NC_10_N) were also made using the same procedure. The electrolyte for all the OECT measurements was 0.1 M NaCl aqueous solution unless otherwise specified. The OECTs were characterized using a Keithley 4200A-SCS.

### SPICE simulation

The SPICE models of K-OECT and Na-OECT were created in B2 SPICE (EMAG Technologies). These models simulate the spiking features of c-OECNs. The details of the simulation are presented in Supplementary Note 4.

### Cuff electrode fabrication and interfacing with the vagus nerve

To interface with the mouse’s vagus nerve, a flexible array of electrodes was fabricated based on a previous protocol^33^. Eight stimulation electrodes (450 µm × 200 µm in dimensions) in a 2 × 4 arrangement were included in the array. The substrate and insulation layers consisted of flexible PaC which allow the device to conformally wrap around the nerve, and thus to serve as a cuff-electrode-style interface. The first stage of the microfabrication process consisted of a cleaning procedure for glass microscope slides using ultrasonication in a dilute Hellmanex soap solution (2 vol% in deionized water), then in acetone, followed by isopropanol. A flexible PaC layer (2 µm) was then deposited using chemical vapour deposition (Diener Electronic) on the glass carrier substrates. Photolithographic patterning of metal interconnects (80 nm gold and 5 nm titanium adhesion layer) was carried out through a lift-off process using the negative photoresist AZ nLof 2070 and a MA6 Suss mask aligner with an i-line filter. These metal patterns provide the electrode contact surfaces and an electrical connection between the electrodes and the back-end contact pads. Following lift-off, a 2 min oxygen plasma process at a power of 50 W was performed before the deposition of an insulating PaC layer (1.5 µm) on the metal electrode lines (with A-174 adhesion enhancer in the deposition chamber). An AZ 10XT photoresist etch mask was patterned, and RIE was performed (O_2_/CF_4_ gases, 150 W) to define the shape of the probes. Acetone and isopropanol rinses were used to remove the photoresist etch mask. Next, a dilute soap antiadhesion layer (2.5 vol% in deionized water) was spin-coated at 1,000 r.p.m. on the sample surface. A thick sacrificial PaC layer (2 μm) was then deposited and the above RIE etch process was used to define the surface of the electrode and back-end contact openings. A conductive polymer electrode coating was spin-coated onto the substrates, consisting of a PEDOT:PSS-based dispersion, 5 wt% ethylene glycol, 0.1 wt% dodecyl benzene sulfonic acid and 1 wt% of (3-glycidyloxypropyl)trimethoxysilane. A soft bake was carried out at 100 °C for 60 s, followed by peel-off of the sacrificial PaC layer. Finally, a 45 min annealing process at 140 °C was used to crosslink the conducting polymer film, and the individual arrays were removed from the glass substrates using deionized water to assist the process.

To provide electrical connection to the arrays, custom adaptors were created using zero-insertion-force clips (ZIF-Clip) mounted on printed circuit boards. Back-end wiring provided the possibility for connection to an Intan 16-channel head stage of an Intan RHS 128 channel stimulation/recording controller (Intan Technologies) with software that helps verify good contact and electrochemical impedance values. When interfacing the c-OECN and the right vagus nerve, four neighbouring electrodes were shorted together and connected to the output of the c-OECN.

C57BL/6J male mice, 10–12 weeks of age, purchased from Charles River Laboratories, were used in experiments investigating the interface with the vagus nerve. Mice were housed under a 12 h light/dark cycle with adequate access to food and water. The approval for this experimental protocol was provided by the regional Stockholm Animal Research Ethics Committee (Stockholm, Sweden). The surgery used to isolate the vagus nerve has been described previously^34^. In brief, mice were anaesthetized with isoflurane and an equal mixture of air and oxygen. They were then placed in the supine position, and a ventral midline cervical incision was made to expose subcutaneous tissues and the mandibular salivary glands. After separation using blunt dissection, proper exposure revealed the right neurovascular bundle containing the cervical vagus nerve and carotid artery. The vagus nerve was dissected from the vasculature and isolated before immobilizing with a suture to facilitate electrode placement.

### Reporting summary

Further information on research design is available in the Nature Portfolio Reporting Summary linked to this article.

## Online content

Any methods, additional references, Nature Portfolio reporting summaries, source data, extended data, supplementary information, acknowledgements, peer review information; details of author contributions and competing interests; and statements of data and code availability are available at 10.1038/s41563-022-01450-8.

## Supplementary information

Supplementary Information Supplementary Notes 1–6, Figs. 1–45 and Tables 1 and 2.

Reporting Summary

Supplementary Video 1 The operation mechanism of the c-OECN showing the states of various components with time.

## Data Availability

The authors declare that the main data supporting the findings of this study are available within the paper and its Supplementary Information files. Source data are provided with this paper.

## Source data

Source Data Fig. 1 to 4 Source data for Figs. 1 to 4 in a single Excel file.
