# Supplementary material for: Ion-tunable antiambipolarity in mixed ion–electron conducting polymers enables biorealistic organic electrochemical neurons
Source: Nat Mater. 2023 Jan 12;22(2):242–8. doi: 10.1038/s41563-022-01450-8 (PMC9894750; doi:10.1038/s41563-022-01450-8)
Supplement: Supplementary file 1 — Supplementary Notes 1–6, Figs. 1–45 and Tables 1 and 2. [file 41563_2022_1450_MOESM1_ESM.pdf]

nature materials

Article https://doi.org/10.1038/s41563-022-01450-8

Ion-tunable antiambipolarity in mixed ion–electron conducting polymers enables biorealistic organic electrochemical neurons

In the format provided by the authors and unedited

Table of Contents

Supplementary Notes

Supplementary Note 1.	Mechanism of the shift in Vp in ammonium ion-based electrolytes and neurotranmitters……………………….	7
Supplementary Note 2.	Stability of the neuron and BBL at high voltages………	19
Supplementary Note 3.	Hodgkin-Huxley Neuron model………………………..	22
Supplementary Note 4.	OECT HH neuron circuit analysis and SPICE simulations………………………………………………	22
Supplementary Note 5.	Power consumption and energy efficiency estimation and downscaling……………………………………...	28
Supplementary Note 6.	Features of neuron……………………………………	32

Supplementary Figures

Supplementary Figure 1.	Stable antiambipolar behavior in BBL showing positive and negative transconductance regions…………………	3
Supplementary Figures 2,3.	Calculation of the number of electrons injected per repeating unit into BBL during doping and reproducibility…………………………………………..	3
Supplementary Figure 4.	Correlation between applied gate voltage and electrons per repeating unit (eru) …………………………………	4
Supplementary Figure 5.	The structure of BBL and the stability of antiambipolar behavior. Structures of PEDOT:PSS, P(g42T-T), P(g7NC10N), and the instability of antiambipolar behavior in these polymers…………………………...	5
Supplementary Figure 6.	Changes in UV-Vis absorption spectra of the polymers under investigation with voltage cycling………………..	6
Supplementary Figure 7.	Instability of the polymers under investigation with voltage cycling under oxygen-free environments………	6
Supplementary Figure 8.	First 10 voltage scans after adding 3.3 mM neurotransmitters to 100mM NaCl, OECT transfer curves in various ammonium salt-based electrolytes (100mM), and structure of the ammonium cations……..	7
Supplementary Figure 9.	Cyclic voltammetry of BBL in various electrolytes…….	8
Supplementary Figures 10-13.	FTIR spectrum of BBL after voltage cycling in NaCl, NH_4Cl, dopamine hydrochloride and guanidinium chloride…………………………………………………	9
Supplementary Figure 14.	The comparison of electrons per monomer unit in the case of 0.1 M NaCl and NH_4Cl………………………	12
Supplementary Figures 15, 16.	The τ_{ON} and τ_{OFF} of Na-OECT and K-OECT for different voltages………………………………………..	13

Supplementary Figure 17.	Inverting amplifier with an NMOS transistor ………….	14
Supplementary Figures 18-25.	The operation mechanism of c-OECN showing the states of various components as time progresses………………	14
Supplementary Figure 26.	PEDOT:PSS OECT-based Inverter configuration and inverter voltage transfer characteristics (VTC)…………	18
Supplementary Figure 27.	Neural features based on all OECT-based circuits……..	19
Supplementary Figure 28.	The transfer curve and the gate current in the BBL OECT showing water splitting at voltages higher than 1.3 V………………………………………………	20
Supplementary Figures 29, 30.	Stability of the BBL OECT on voltage cycling ……….	20
Supplementary Figure 31.	Stability of the c-OECN ………………………………...	21
Supplementary Figure 32.	Symbol of the SPICE model for the BBL n-type OECTs with antiambipolar/Gaussian characteristics…………….	24
Supplementary Figure 33.	Comparison of simulated and measured transfer characteristics of Na and K-OECTs……………………..	24
Supplementary Figure 34.	A typical tonic spiking c-OECN with SPICE models…...	25
Supplementary Figure 35.	Neural features based on SPICE simulations with K-OECT and Na-OECT models…………………………...	25
Supplementary Figures 36-38.	c-OECN with an NMOS transistor to replace the K-OECT and experimental and simulated neural features…	26
Supplementary Figure 39, 40.	Power and energy consumption of c-OECN and its scaling…………………………………………………	31
Supplementary Figure 41.	The inhibition of spiking using GABA.	33
Supplementary Figure 42.	Transfer characteristics of the Na-OECTs and K-OECTs used in the circuit ………………………………………	34
Supplementary Figure 43.	Interfacing the cuff electrodes with the right vagus nerve. …………………………………………………	34
Supplementary Figures 44,45.	Photographs of the c-OECN circuit……………………..	35

Supplementary Tables

Supplementary Table 1.	Parameters used for different configurations of c-OECN.	36
Supplementary Table 2.	Comparison of artificial neurons based on various technologies……………………………………………...	37

References

37

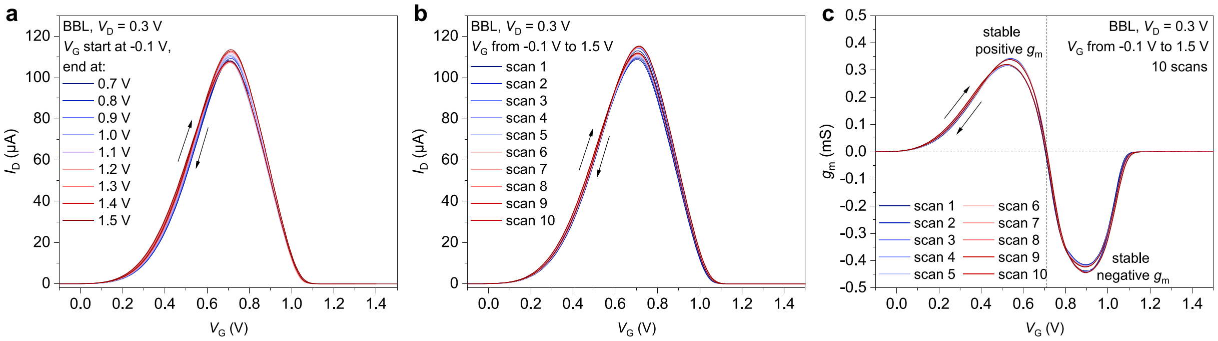

Supplementary Figure 1. (a, b) stable antiambipolar behavior in BBL showing (c) positive and negative transconductance regions.

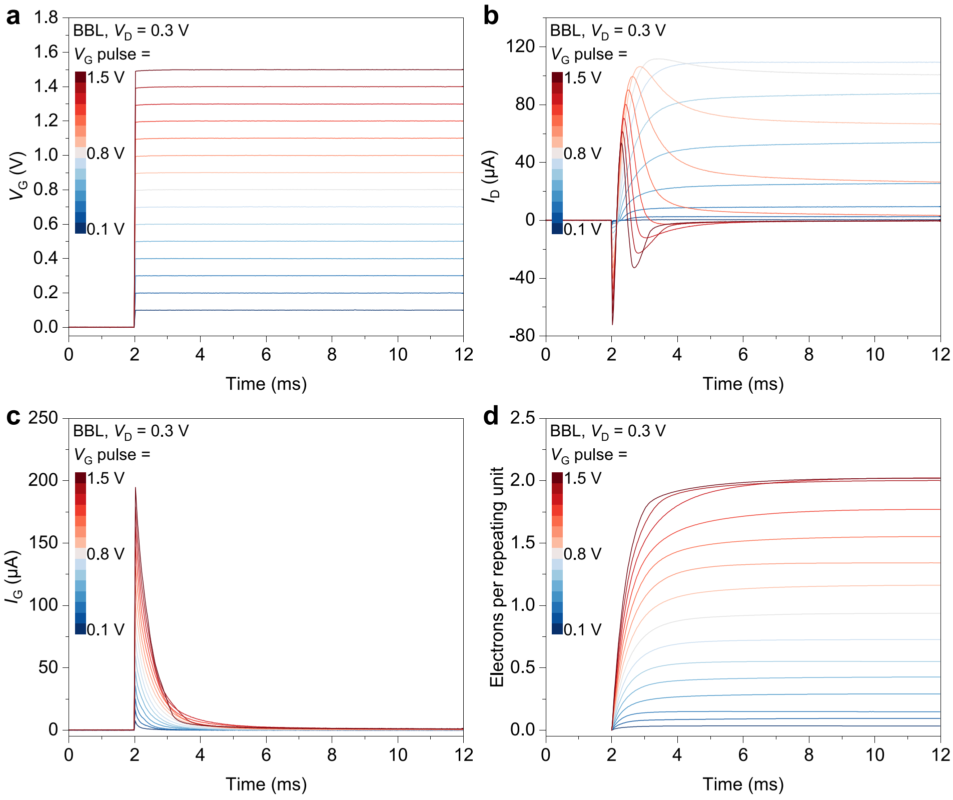

Supplementary Figure 2. Calculation of the number of electrons injected per repeating unit into BBL during doping. (a) Applied gate voltage over 12 ms, (b and c) the corresponding drain and gate currents. (d) The number of electrons per repeating unit (eru) is obtained by integrating the gate current over time using the relation:

$$eru = \frac{\int_{t_1}^{t_2} I_G \, dt}{F} \cdot \frac{M_{mol}}{\rho V}$$

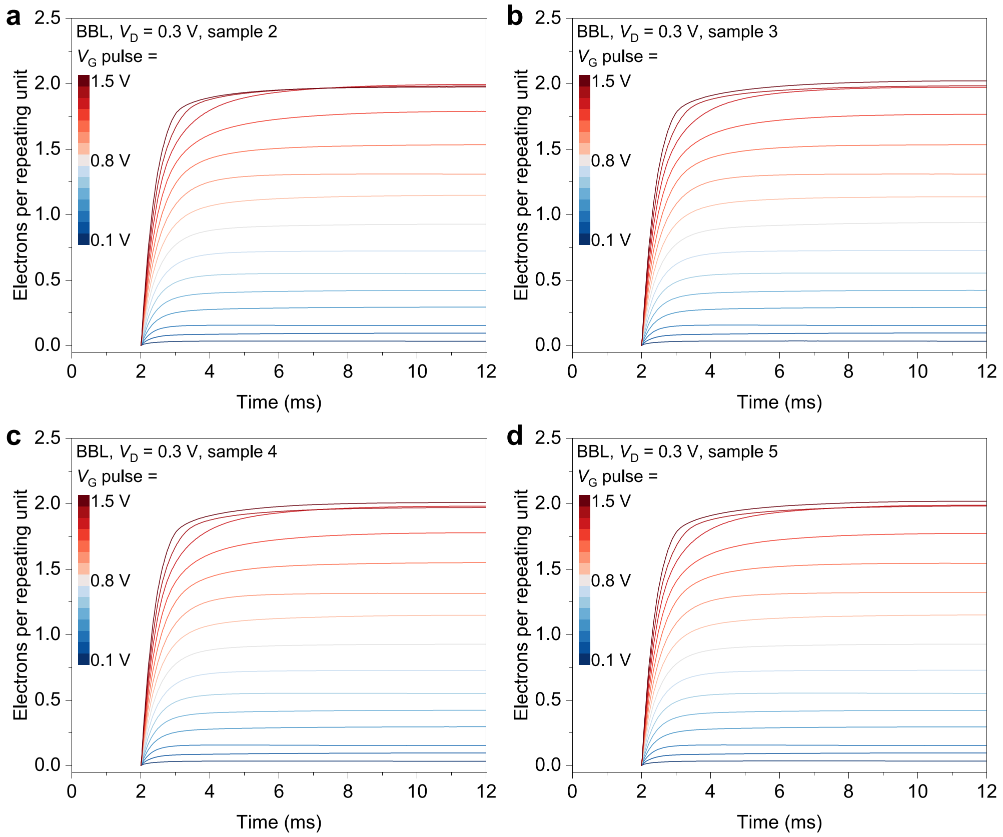

Supplementary Figure 3. (a-d) Reproducibility of the calculation of electrons injected per repeating unit across four samples.

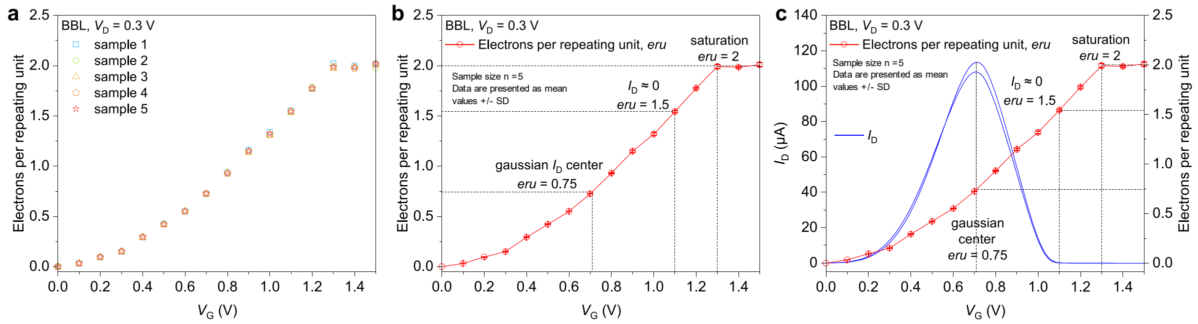

Supplementary Figure 4. (a-c) Correlation between applied gate voltage and electrons per repeating unit (eru) at a drain voltage of 0.3 V and the reproducibility across various samples. The measurements were taken on 5 distinct samples.

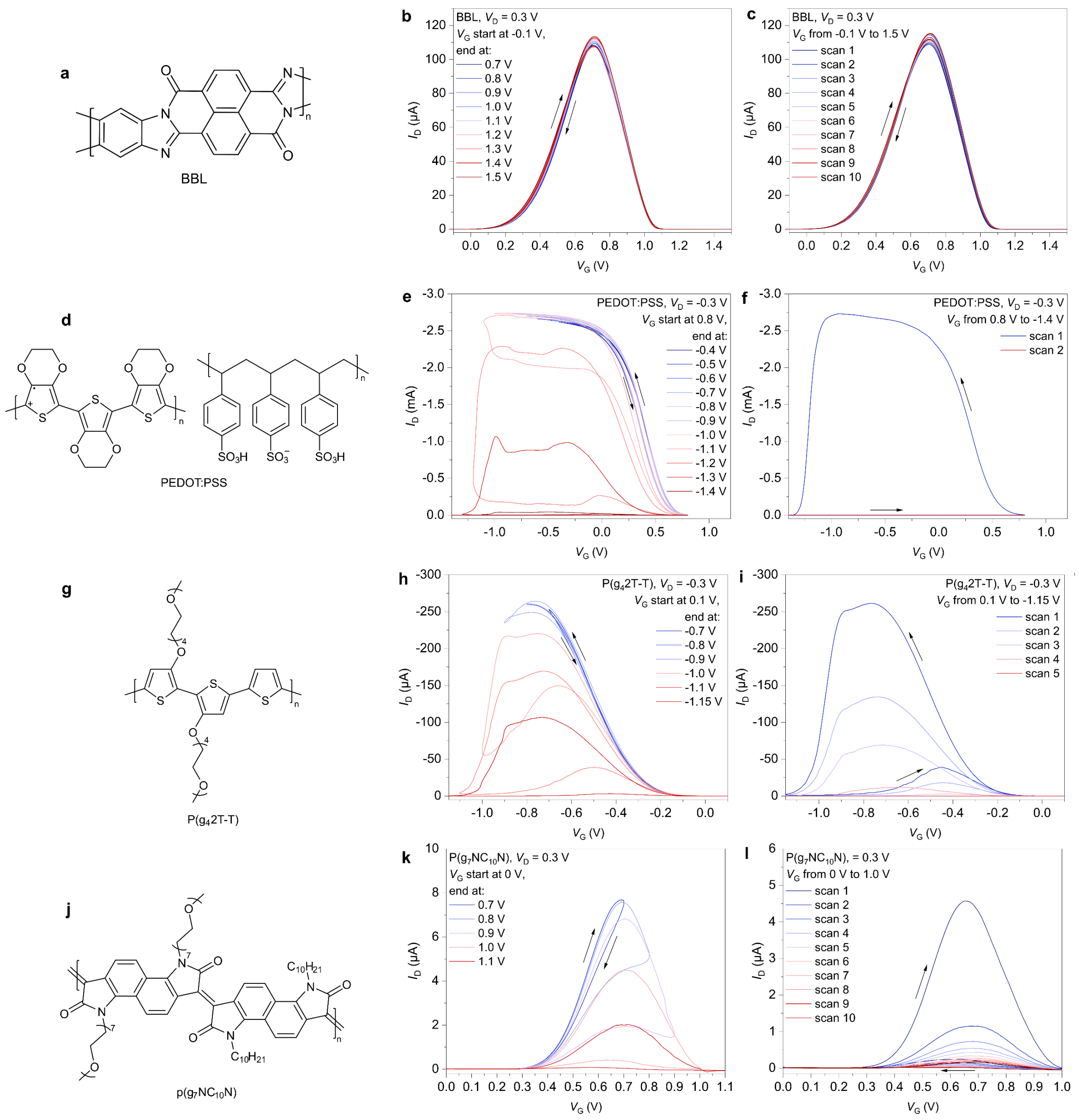

,

Supplementary Figure 5. (a-c) The structure of BBL and the stability of antiambipolar behavior. (d-k) Structures of PEDOT:PSS, P($g_4$2T-T), P($g_7NC_{10}N$), and the instability of antiambipolar behavior in these polymers. P($g_4$2T-T) is dissolved in 1,2-dichlorobenzene and p($g_7NC_{10}N$) in chloroform and spin-coated (2000 rpm, 60 s, acceleration 2000 rpm s^{-1}) on OECT substrates.

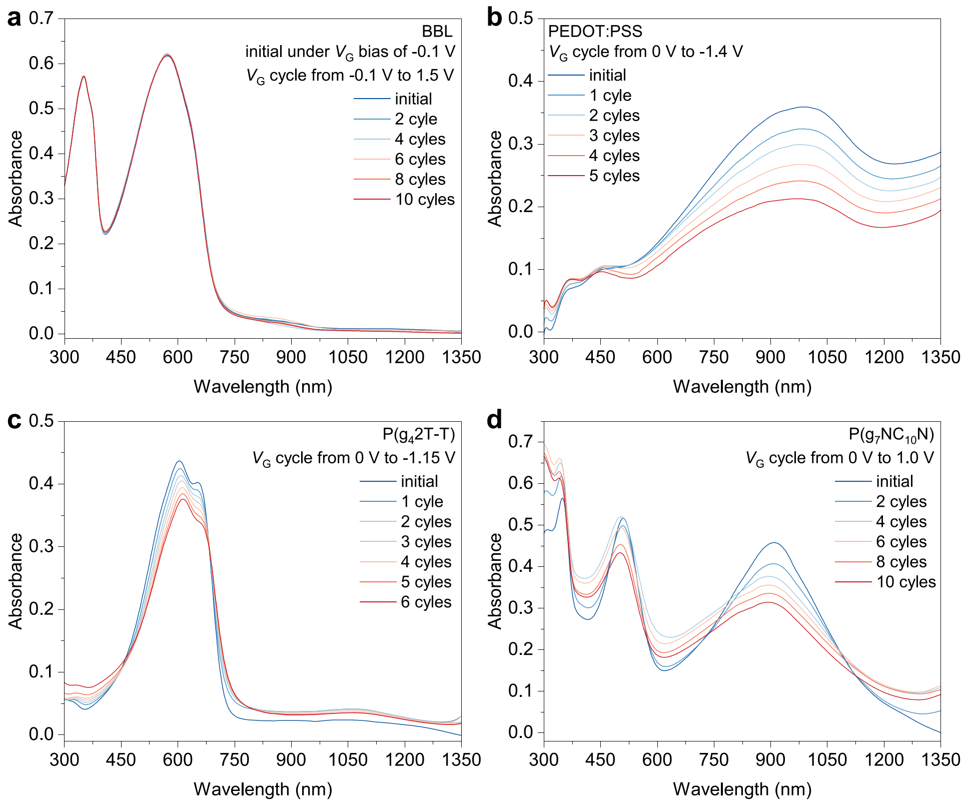

Supplementary Figure 6. Changes in UV-Vis absorption spectra of the polymers under investigation with voltage cycling.

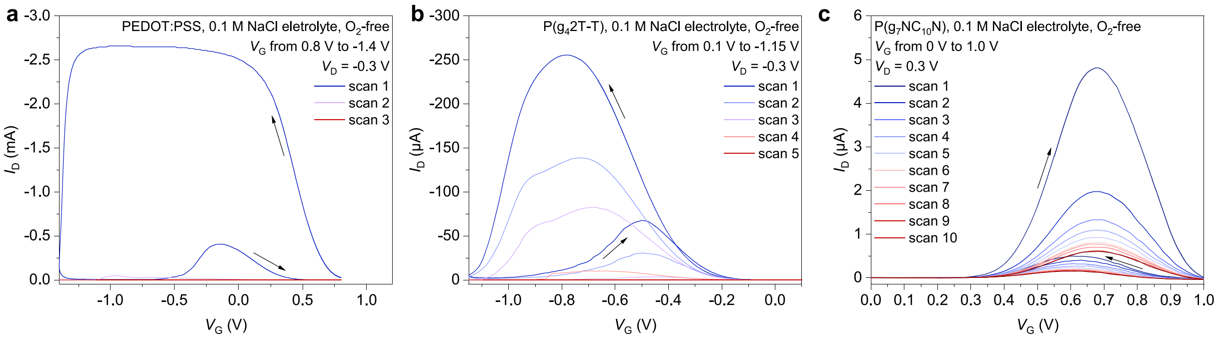

Supplementary Figure 7. Instability of the polymers under investigation with voltage cycling under oxygen-free environments.

Supplementary Note 1. Mechanism of the shift in V_P in ammonium ion-based electrolytes and neurotransmitters.

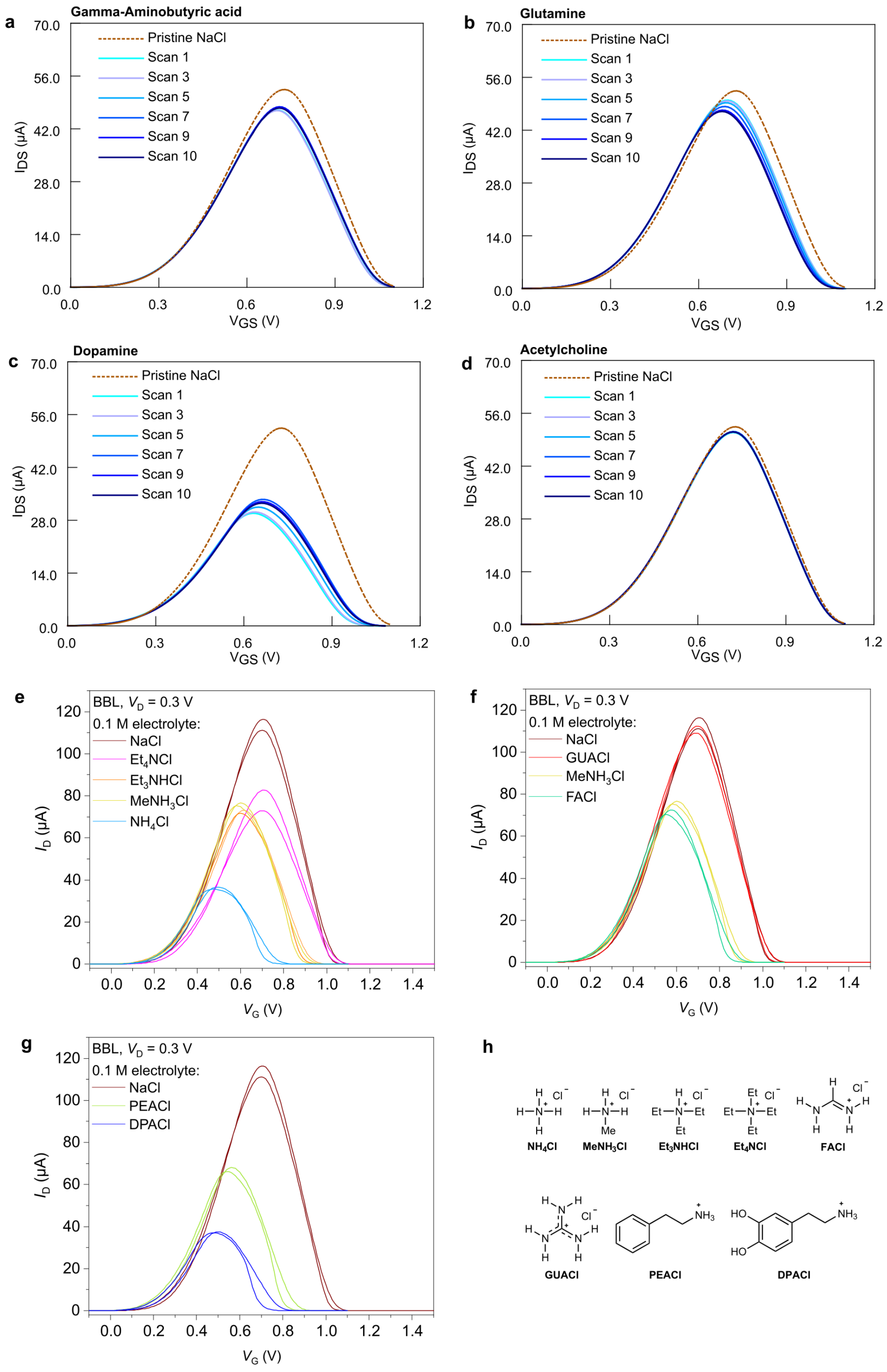

Supplementary Figure 8. (a-d) First 10 voltage scans after adding 3.3 mM neurotransmitters to 100mM NaCl. (e-g) OECT transfer curves in various ammonium salt-based electrolytes (100mM). (h) Structure of the ammonium cations.

The origin of neurotransmitters affecting the V_P of the antiambipolar OECT is similar to the effect caused by NH_4^+. We observe that the OECT can be operated entirely with NH_4^+ and other ammonium ion-based electrolytes. The shift in V_P (ΔV_P) also happens when using other ammonium ions capable of hydrogen bonding, indicating such interactions as the potential origin of the shift. ΔV_P increases with the number of hydrogen atoms (capable of forming hydrogen bonds) attached to the nitrogen. For example, as shown in **Supplementary Figure 8**, ΔV_P increases in the order tetraethylammonium chloride (0 H, no shift) < triethylammonium chloride (1 H) < methylammonium chloride (3 H) < ammonium chloride (4 H, maximum shift).

However, the effect is not merely dependent on the number of hydrogen atoms but also on the strength/possibility of hydrogen bond formation. This is evident from the lack of shift in the case of guanidinium having the highest number of H attached to N. This happens because the electronegativity on the nitrogen is canceled out due to the relative orientations of nitrogen atoms[1], thus reducing the strength of the hydrogen bond formation. This results in zero dipole moment in guanidinium ions.

Similarly, in neurotransmitters and amino acids, the shift follows a similar trend, with acetylcholine (0 H attached to N) having the least hydrogen bond forming capability showing no shift and dopamine with maximum hydrogen bonding capability resulting in maximum shift **(Figure 1h)** when added to NaCl electrolyte. It should be noted that the OECT can operate entirely in dopamine as well (**Supplementary figure 8g**), with the transfer curves overlapping with NH_4^+. To preclude redox reactions as a potential origin of the shift, we show that phenylethylammonium – PEACl (a molecule similar to dopamine except for the absence of redox-active OH groups) can also shift the V_P significantly.

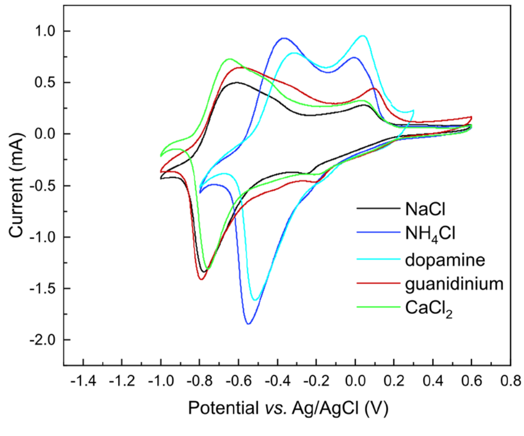

Supplementary Figure 9. Cyclic voltammetry of BBL in various electrolytes.

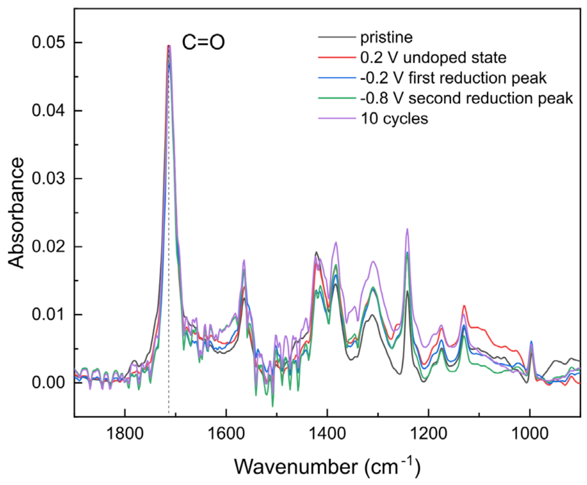

Supplementary Figure 10. FTIR spectrum of BBL after voltage cycling in NaCl.

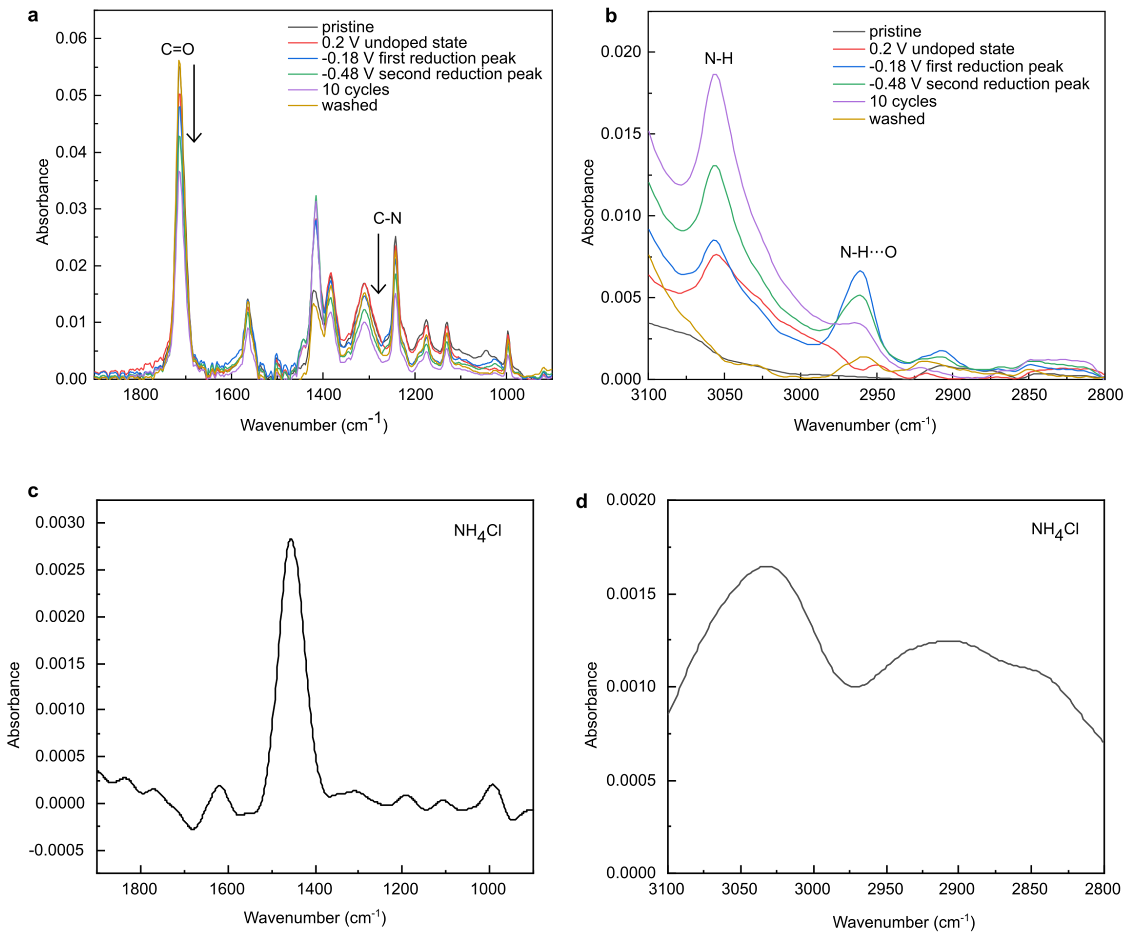

Supplementary Figure 11. (a,b) FTIR spectrum of BBL before and after voltage cycling in NH_4Cl. (c,d) FTIR spectra of NH_4Cl.

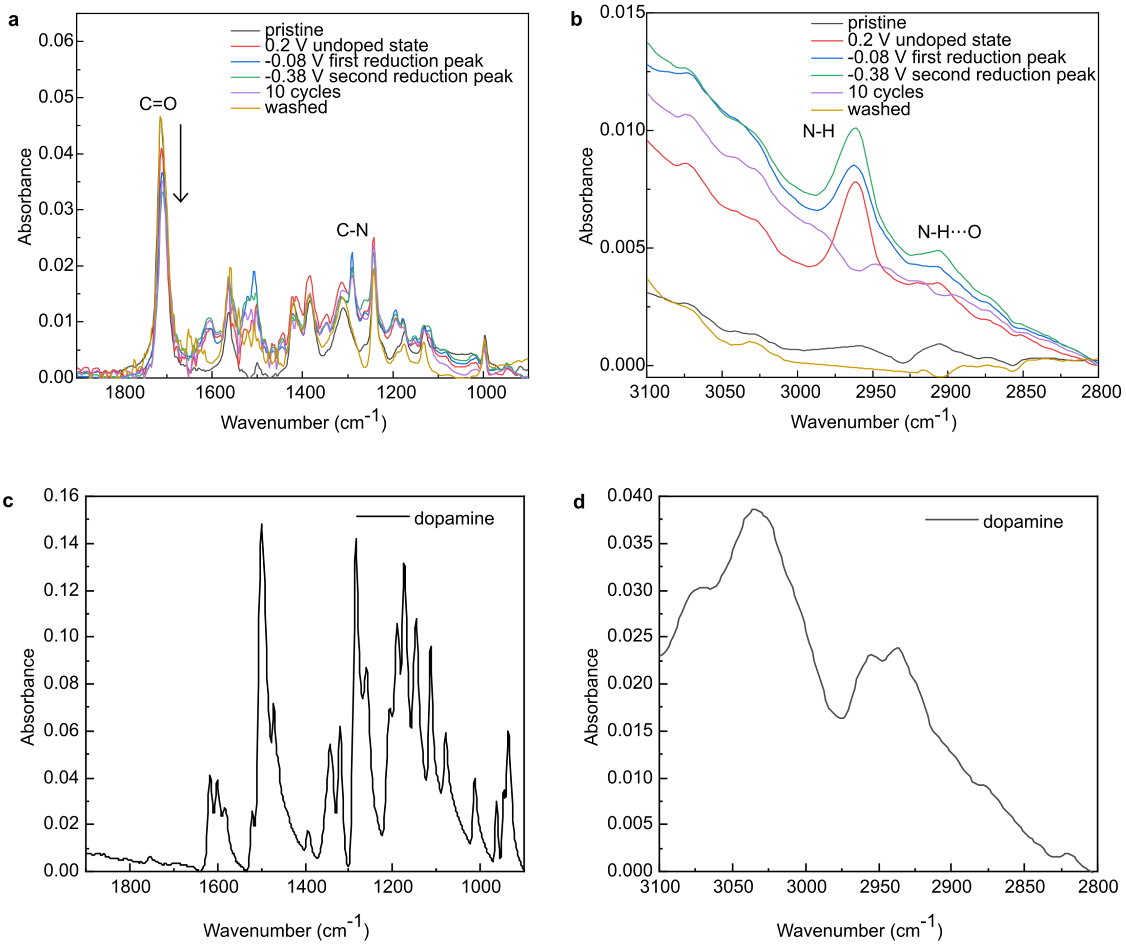

Supplementary Figure 12. (a,b) FTIR spectrum of BBL before and after voltage cycling in dopamine hydrochloride. (c,d) FTIR spectra of dopamine hydrochloride.

The hydrogen in ammonium ions and dopamine could interact with the BBL carbonyl through hydrogen bonding similar to previous reports on NH_4^+ interaction with organic frameworks[2]. This interaction is validated using ex-situ FTIR spectra before and after voltage cycling in the NH_4Cl and dopamine-based electrolytes (**Supplementary Figures 10-12**). FTIR spectra of pristine BBL are taken first. This is followed by applying voltages corresponding to the first and second reduction potentials of BBL obtained from cyclic voltammetry (**Supplementary Figure 9**). BBL is also cycled 10 times in this potential window, and corresponding FTIR spectra for each case are obtained. Cycling in NaCl does not indicate any change in this spectrum. However, when BBL is voltage cycled in NH_4^+, the C=O peaks (1712 cm^{-1}) and C-N (1200 to 1380 cm^{-1}) decrease in amplitude. This is reversed on measuring the BBL in an

undoped state (0.2 V) or after washing the films. Such reversible change in the C=O and C-N indicates an interaction of NH_4^+ ions with the carbonyl group and the nitrogen in BBL. A similar decrease in C=O and C-N signal is also observed in the case of dopamine pointing to similar interactions. Furthermore, the vibration at 2950 cm^{-1} originates from the hydrogen-bonded H atoms' (N-H--O, N-H--N) stretching mode. This intensity increases on applying a reducing potential to BBL (in both NH_4^+ and dopamine) and reverses on washing the film confirming hydrogen bond formation.

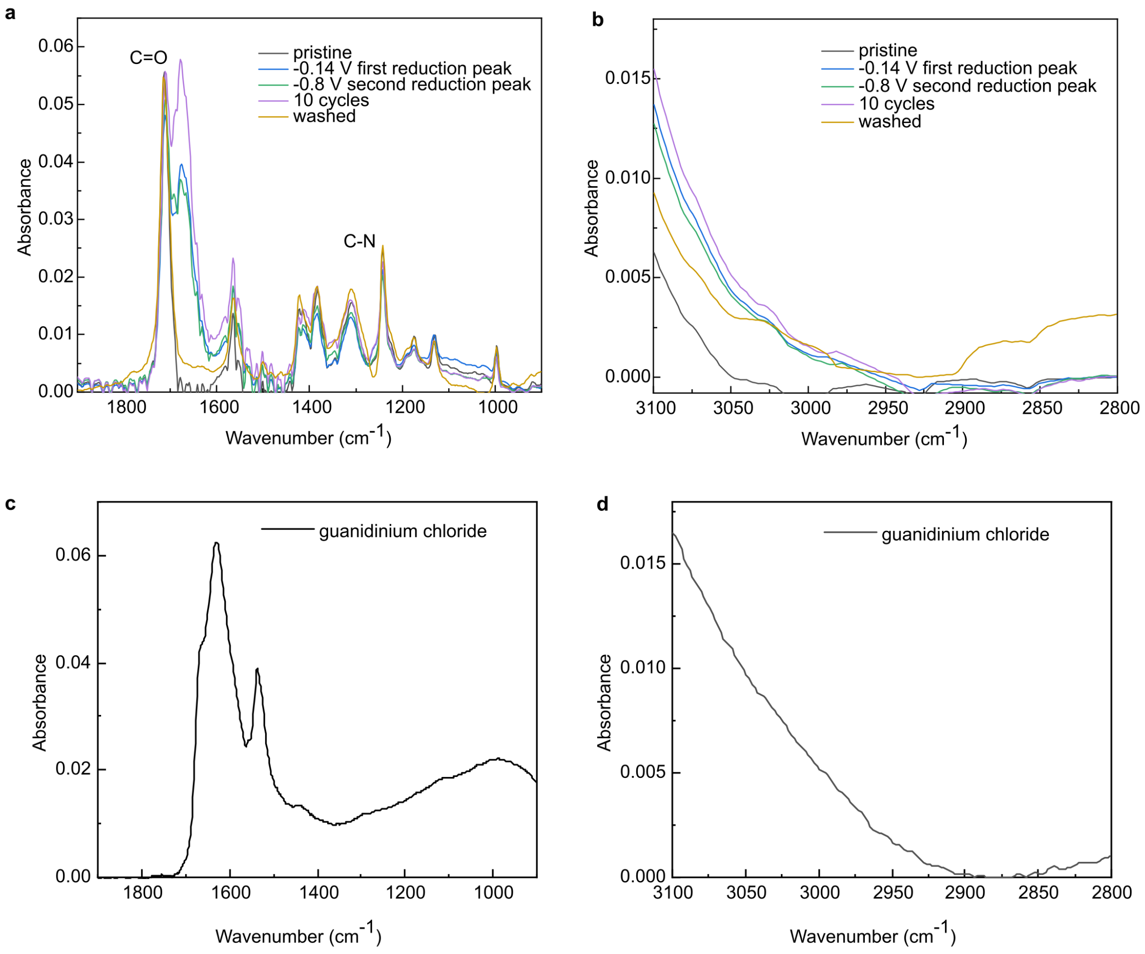

Supplementary Figure 13. (a,b) FTIR spectrum of BBL before and after voltage cycling in guanidinium chloride. (c,d) FTIR spectra of guanidinium hydrochloride.

In the case of guanidinium (**Supplementary Figure 13**), the C=O and C-N signals do not decrease, nor does the vibration at 2950 cm^{-1} increase, indicating the absence of hydrogen bond interactions. This is in line with the lack of V_P shift in the case of guanidinium and confirms the hypothesis that the V_P shift originates from the interaction of NH_4^+/neurotransmitter with C=O and nitrogen of BBL via hydrogen bond. Similar hydrogen bonding interaction between amines and C=O group in polymer has been utilized to enable sensing in previous reports[3]. It has also been known that NH_4^+ can enable high-charge densities in covalent organic frameworks due to H-bonding[2]. In our case, we observed that the electrons injected per monomer unit are much higher in the case of NH_4^+ compared to Na^+ for a given gate voltage. This causes the multiply charged species to be formed at a lower voltage in the case of NH_4^+, thus shifting the V_P to lower values (**Supplementary Figure 14**).

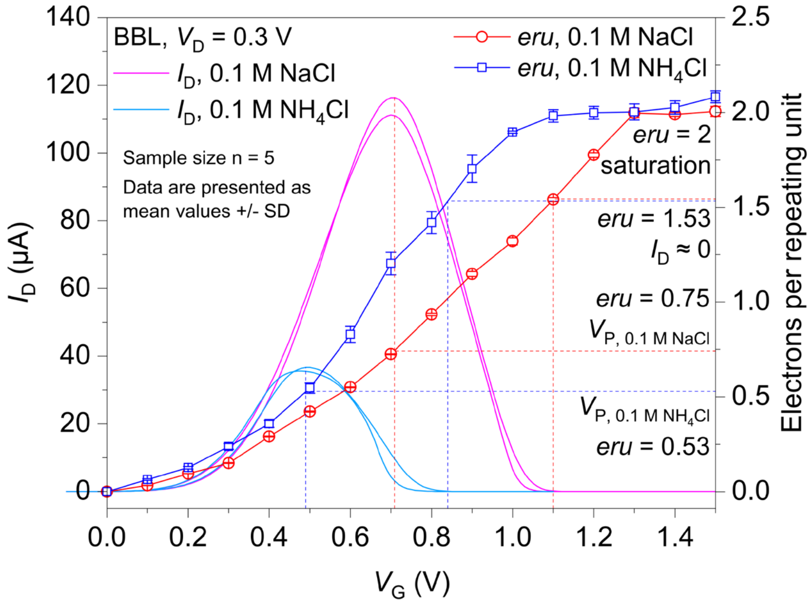

Supplementary Figure 14. The comparison of electrons per monomer unit in the case of 0.1 M NaCl and NH_4Cl. The eru measurements are taken on 5 distinct samples.

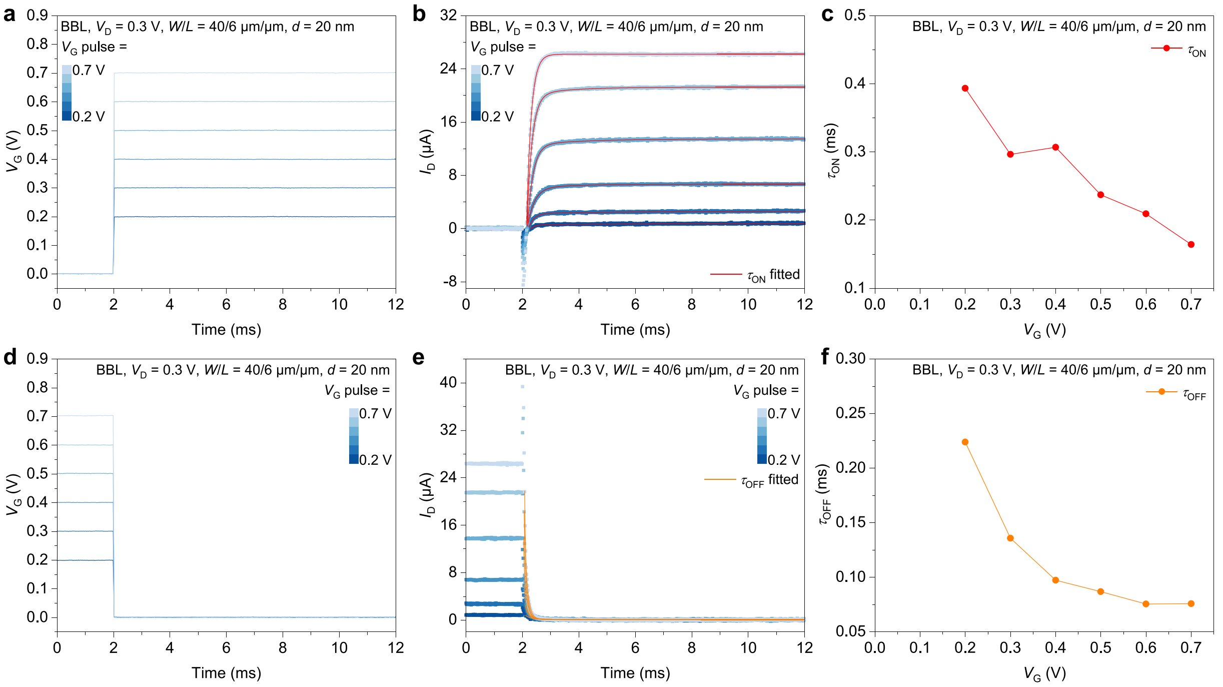

Supplementary Figure 15. (a-c) The τ_{ON} and (d-f) τ_{OFF} of Na-OECT for different voltages.

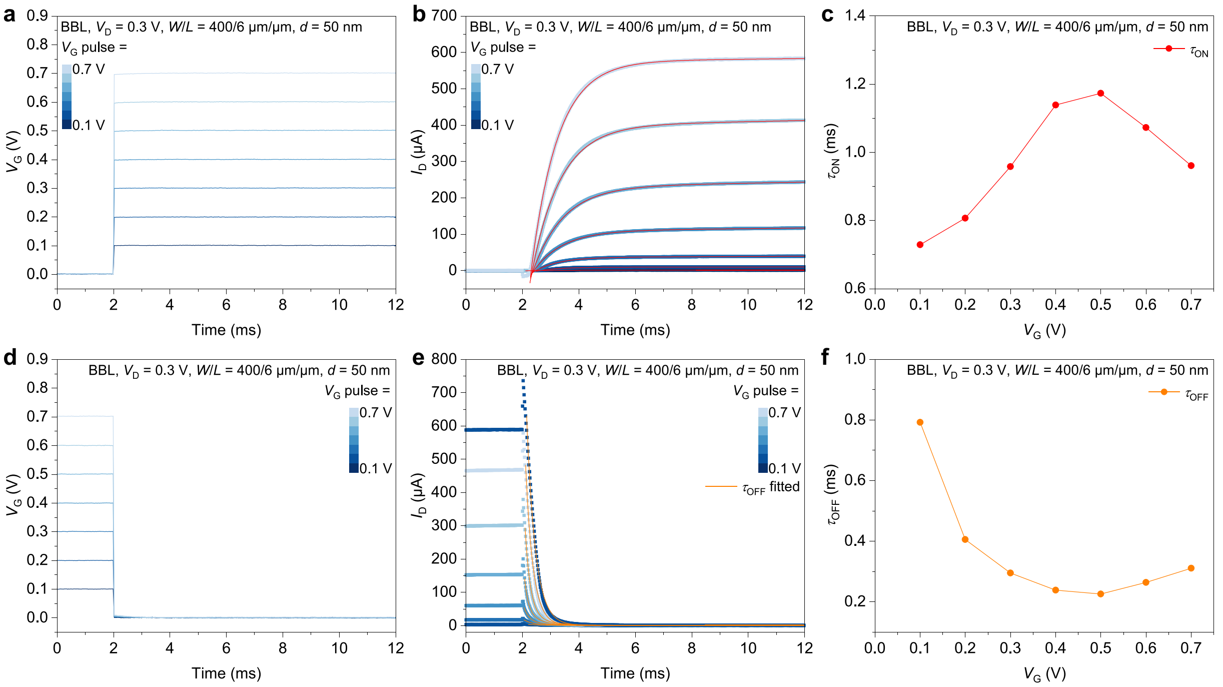

Supplementary Figure 16. (a-c) The τ_{ON} and (d-f) τ_{OFF} of K-OECT for different voltages.

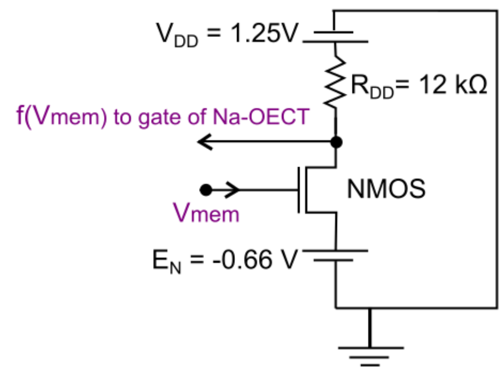

Supplementary Figure 17. Inverting amplifier with an NMOS transistor (Infineon BSP295) and a load resistor (R_{DD}).

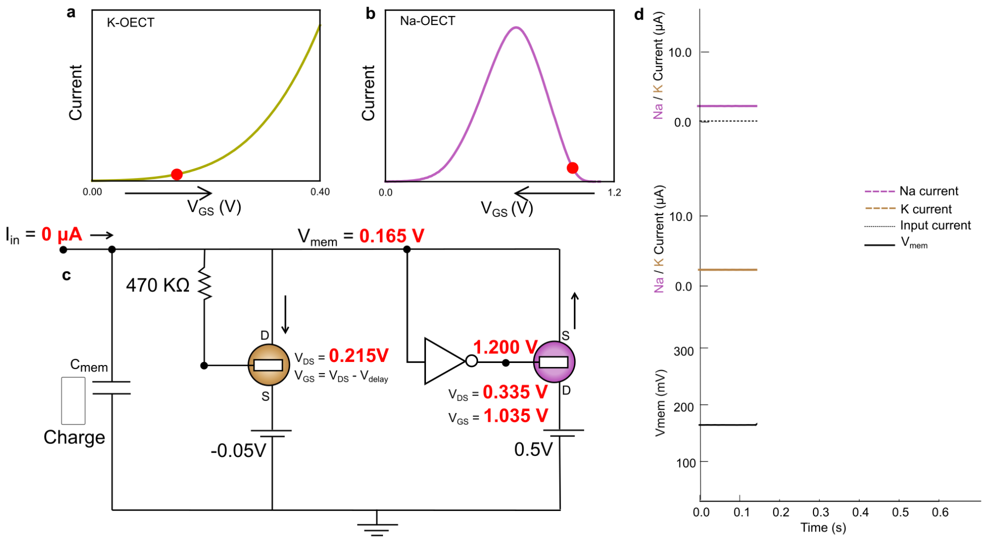

Supplementary Figure 18. The operation mechanism of c-OECN showing the states of various components at t_0.

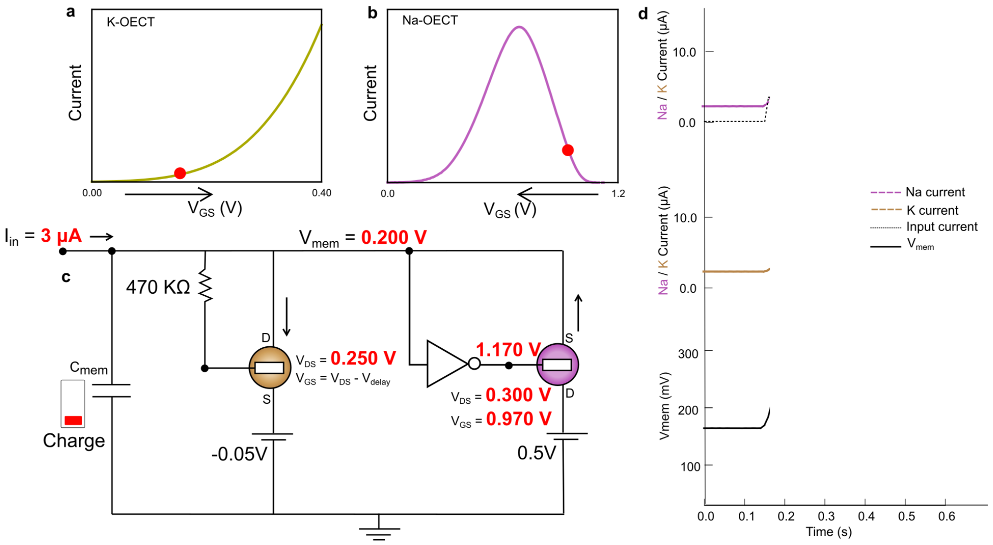

Supplementary Figure 19. The operation mechanism of c-OECN showing the states of various components at t_1. The input current charges the Cmem leading to an increase in V_{mem}.

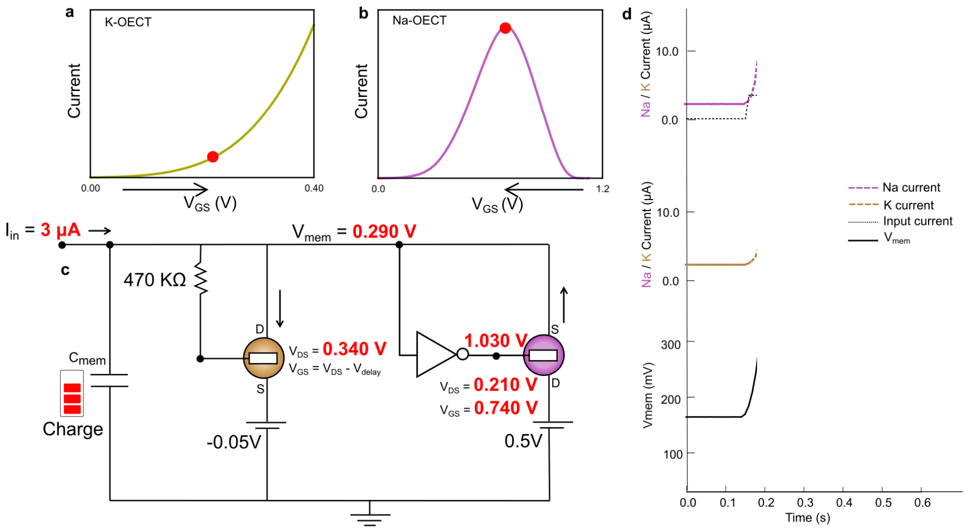

Supplementary Figure 20. The operation mechanism of c-OECN showing the states of various components at t_2. The Na-OECT turns on leading to a rapid increase in V_{mem} which causes depolarization.

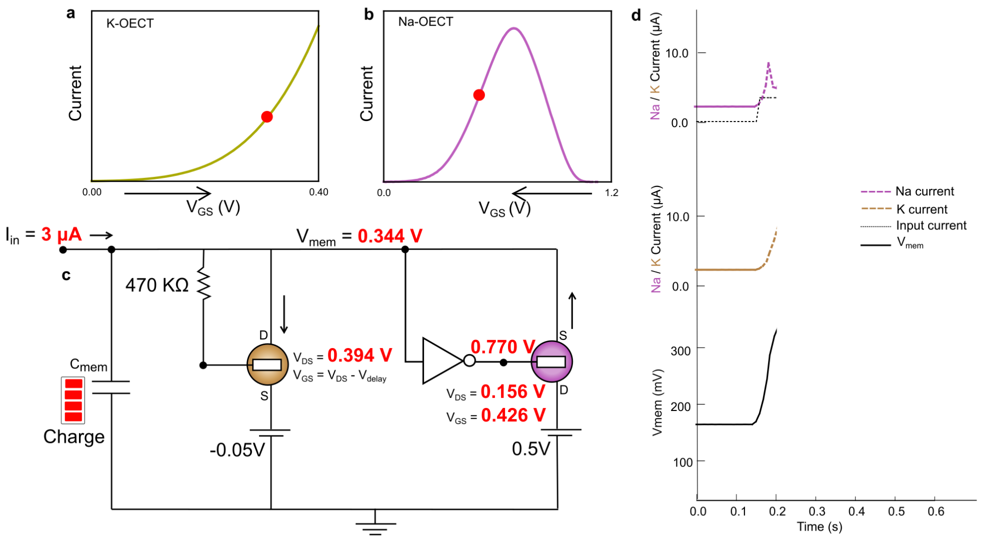

Supplementary Figure 21. The operation mechanism of c-OECN showing the states of various components at t_3. The Na-OECT crosses the maximum current and hence the Na-current drops. The K-OECT turns on after the delay.

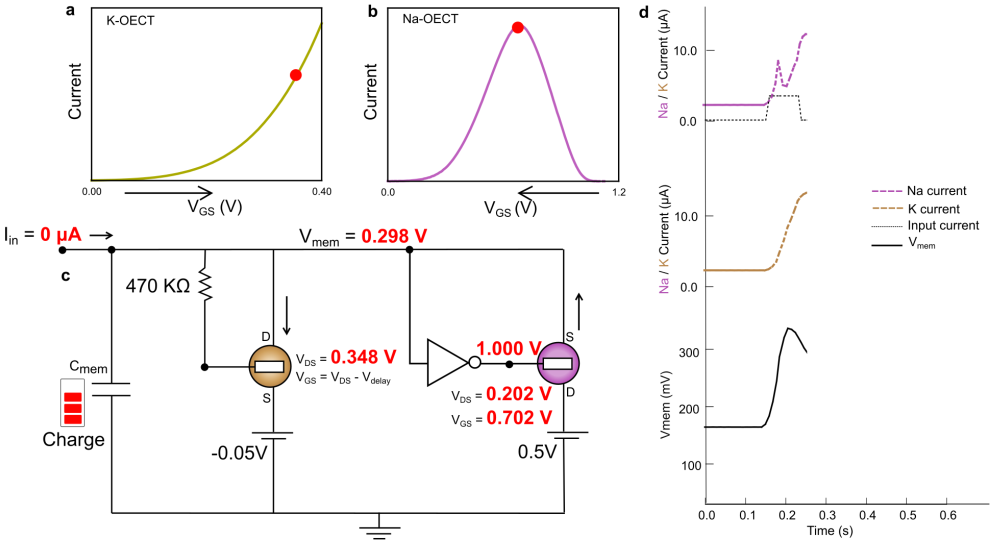

Supplementary Figure 22. The operation mechanism of c-OECN showing the states of various components at t4. The Na-OECT traverses back through V_P leading to a rise in Na-current and creating the second spike in Na. The K-OECT is fully ON and discharges this Na spike completely leading to a reduction in V_{mem} (repolarisation).

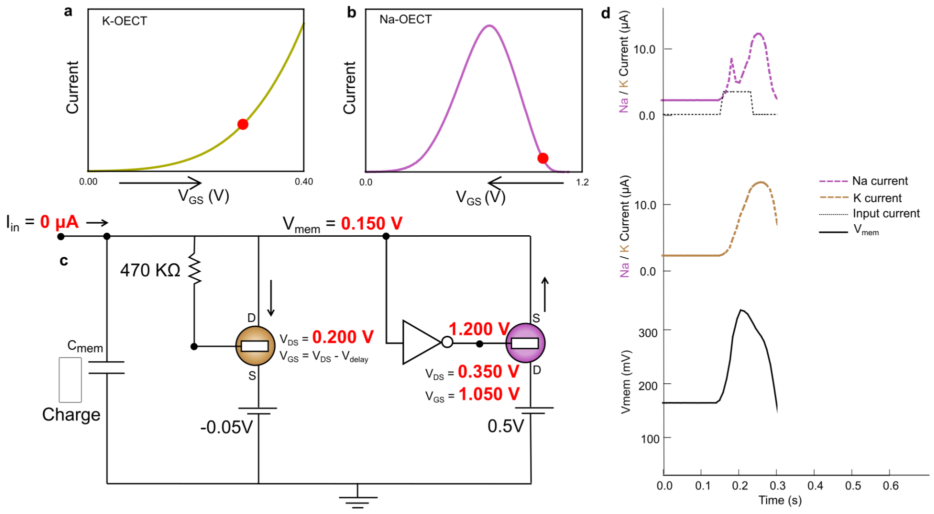

Supplementary Figure 23. The operation mechanism of c-OECN showing the states of various components at t_5. The Na-OECT is back to the OFF state while the K-OECT remains ON, discharging the capacitor completely and bringing the V_{mem} below the baseline causing hyperpolarisation

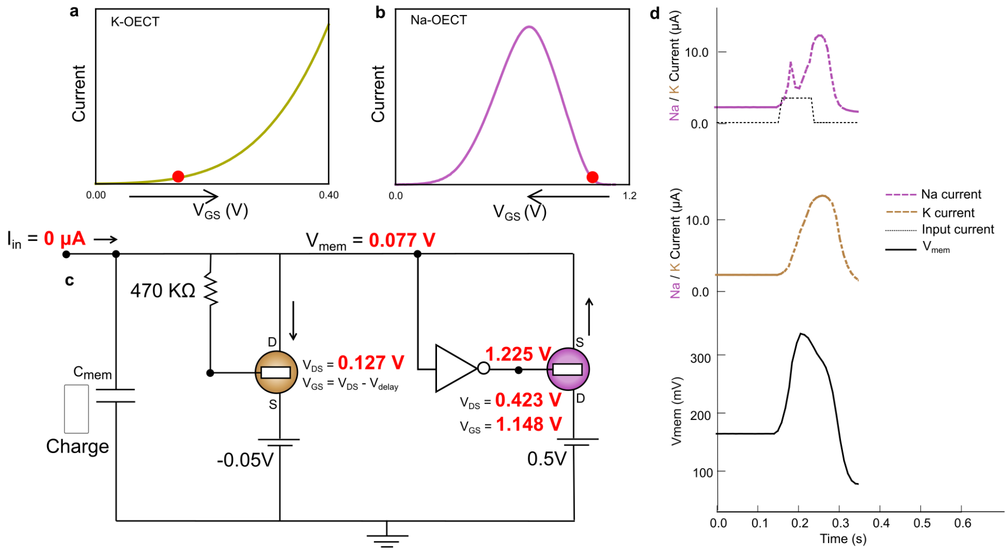

Supplementary Figure 24. The operation mechanism of c-OECN showing the states of various components at t_6. The Na-OECT is in the OFF state, the K-OECT returns to the OFF state. The c-OECN remains hyperpolarized.

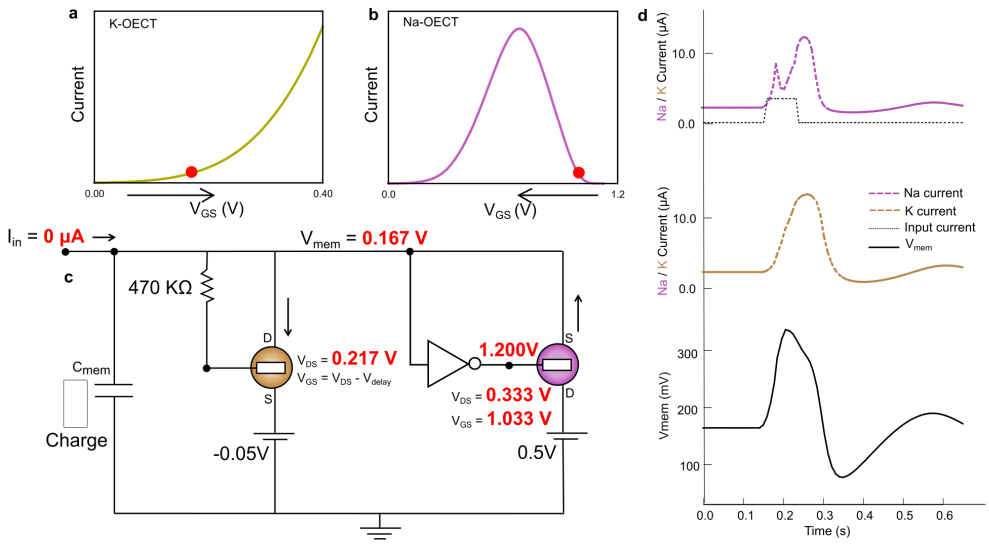

Supplementary Figure 25. The operation mechanism of c-OECN showing the states of various components at t_7. The OECTs return to initial state, and the V_{mem} returns back to the baseline.

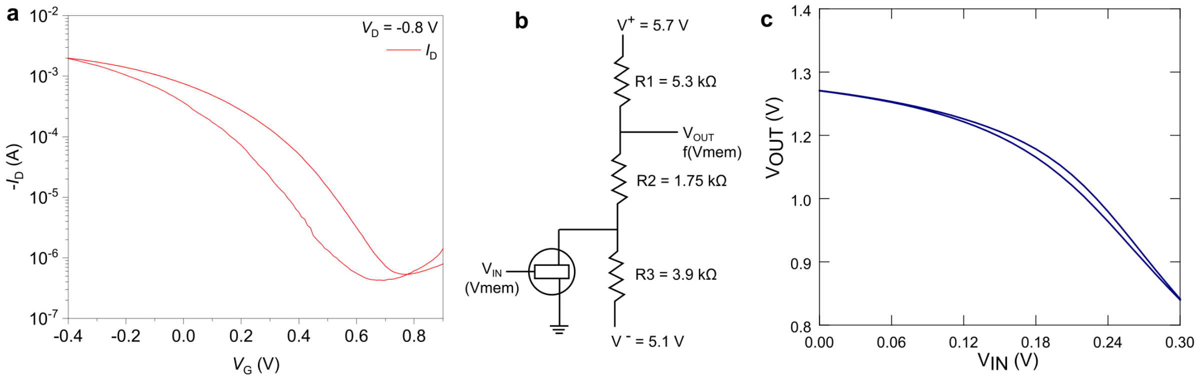

Supplementary Figure 26. (a) Transfer characteristics of PEDOT:PSS OECT. (b) Inverter configuration and (c) inverter voltage transfer characteristics (VTC).

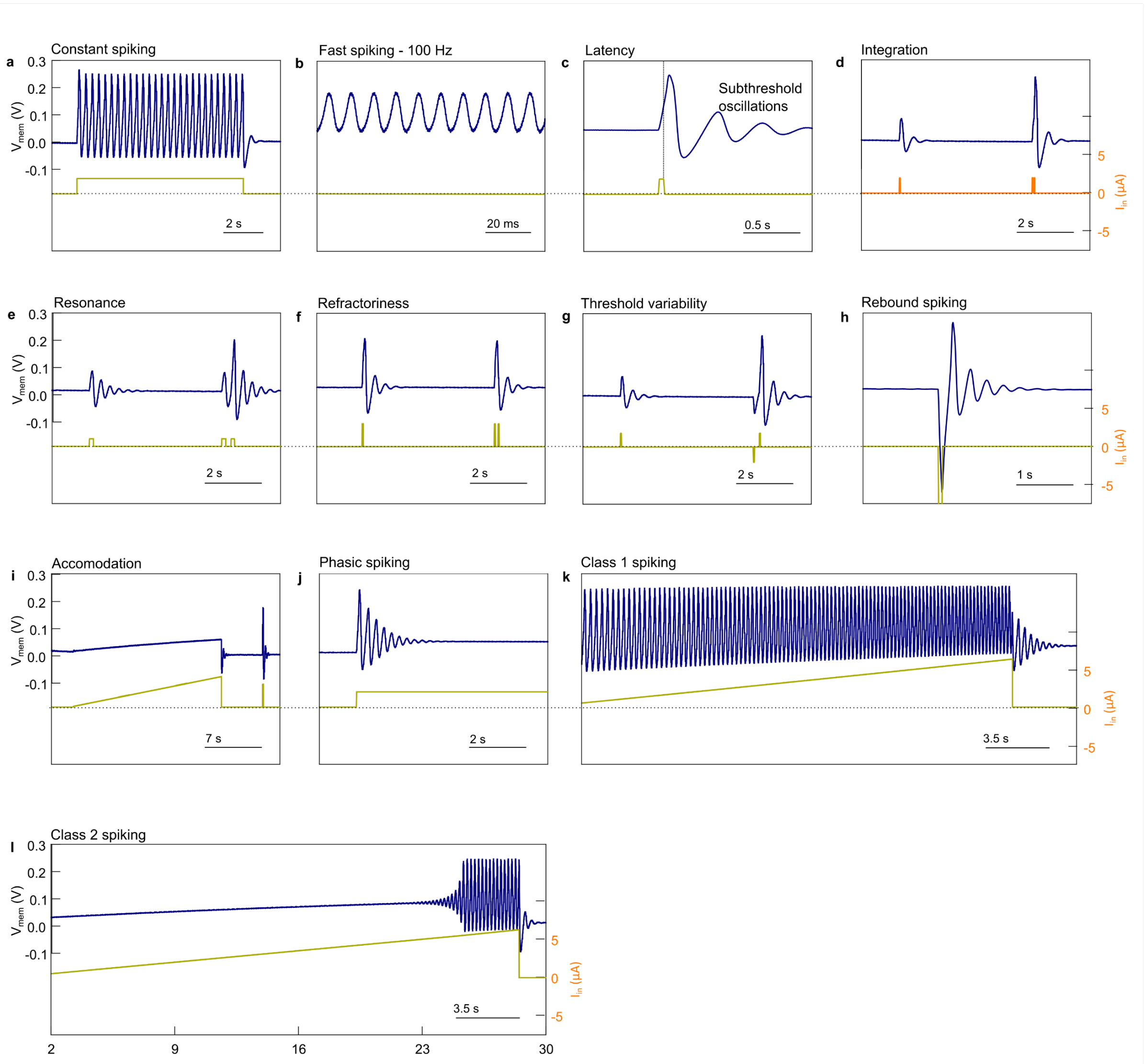

Supplementary Figure 27. Neural features based on all OECT-based circuits.

Supplementary Note 2. Stability of the neuron and BBL at high voltages.

We observed water electrolysis above 1.3 V, as evident from the high gate leakage current (**Supplementary Figure 28**). However, applying such a high voltage did not affect the stability of the OECT considerably (**Supplementary Figures 29** and **30)**. Furthermore, in the neuron, the voltages were adjusted such that the maximum gate voltage on the gate of the BBL OECT is always less than 1.2 V to avoid water electrolysis. Furthermore, we tested the stability of the neuron under continuous operation for around 45 minutes and found no significant change in its spiking behavior (**Supplementary Figure 31**).

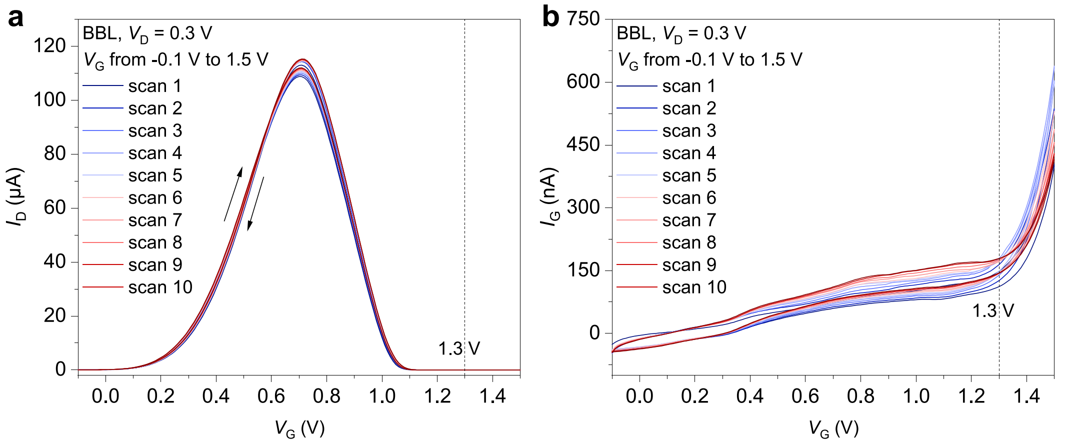

Supplementary Figure 28. (a) The transfer curve and (b) the gate current in the BBL OECT showing water splitting at voltages higher than 1.3 V.

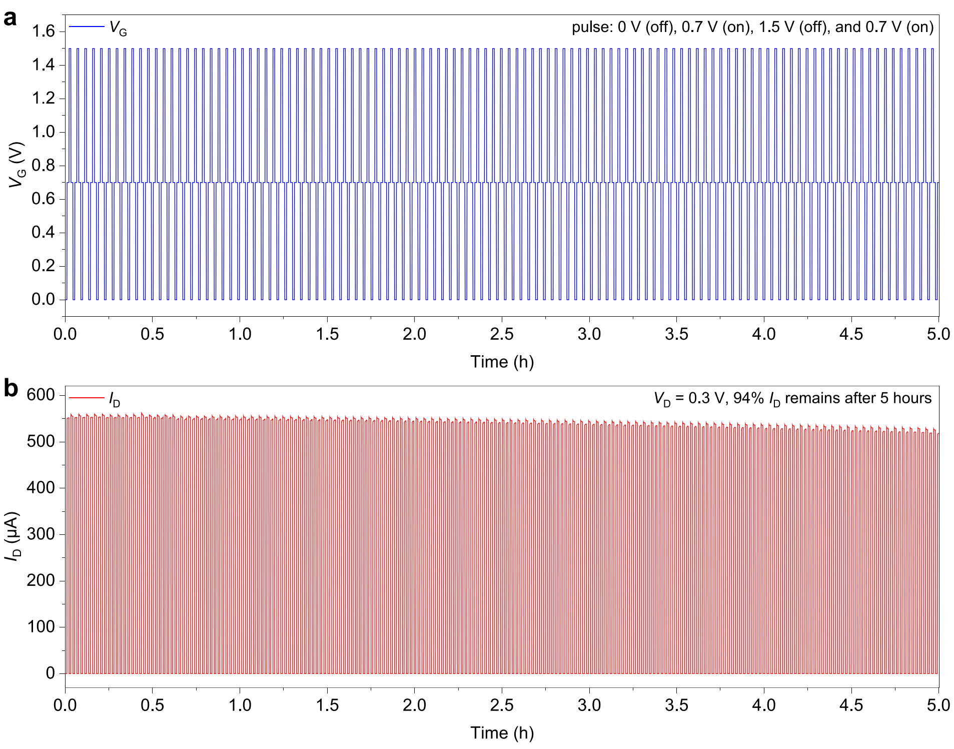

Supplementary Figure 29. (a,b) Stability of the BBL OECT on cycling from 0 (OFF) to 0.7 V (ON) and 1.5 V (OFF).

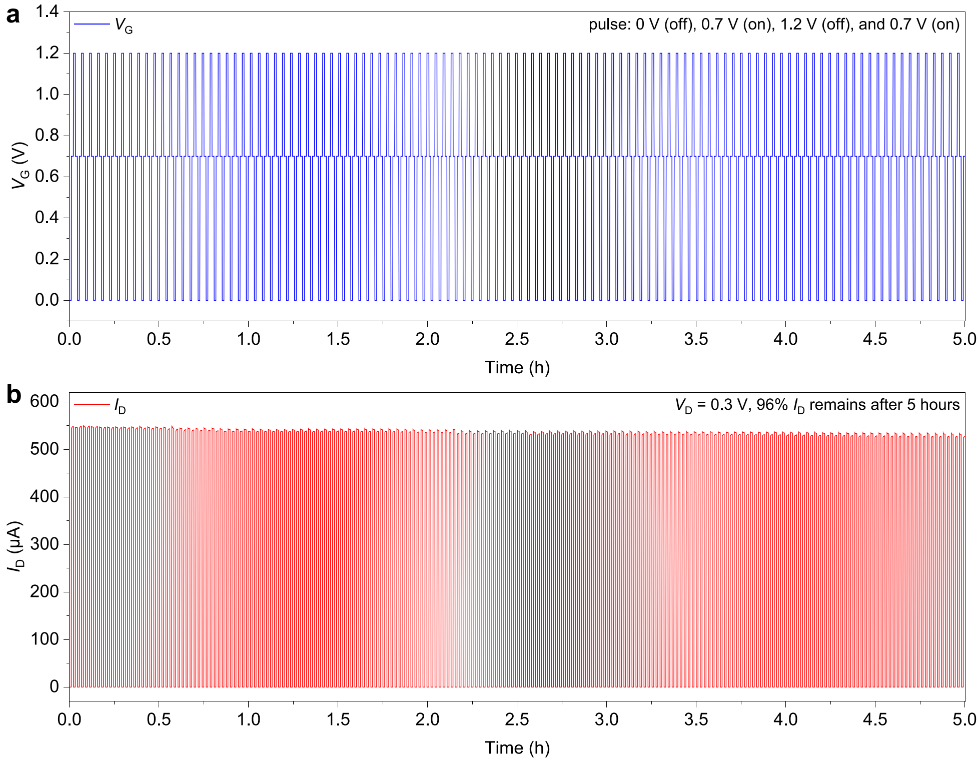

Supplementary Figure 30. (a,b) Stability of the BBL OECT on cycling from 0 V (OFF) to 0.7 V (ON) and 1.2 V (OFF).

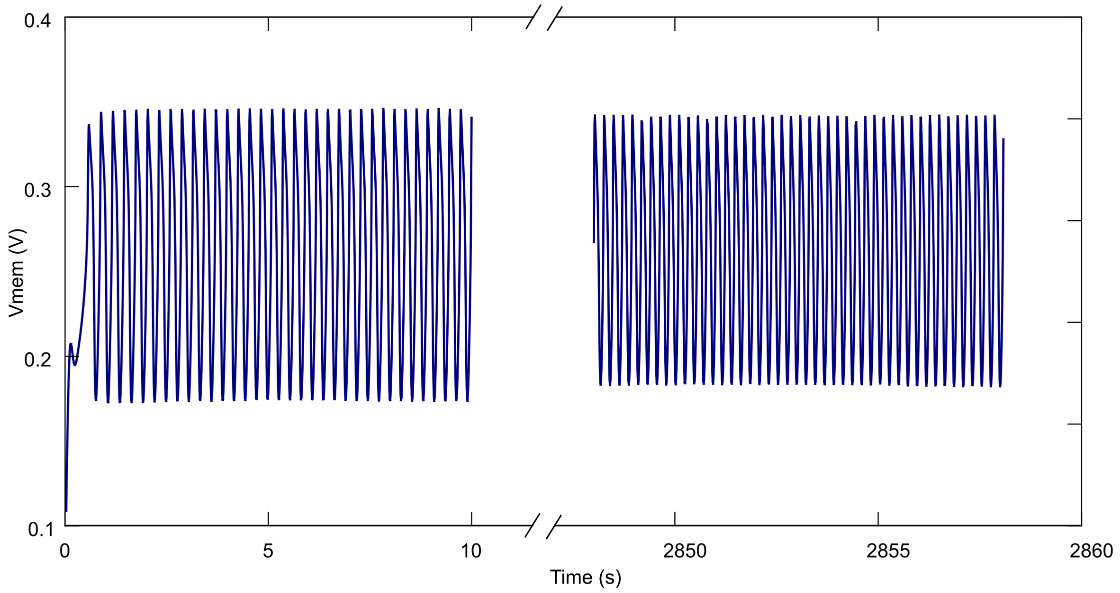

Supplementary Figure 31. Stability of the c-OECN after 45 minutes of continuous spiking.

Supplementary Note 3. Hodgkin-Huxley Neuron model.

The spiking in a neuron is caused by the opening and closing of voltage-gated Na and K ion channels. There is a concentration difference of ions inside and outside neurons maintained by ion pumps. This is modeled as Na and K batteries and forms the lower and upper limits of the action potential. When a current is injected, the membrane capacitance C_{mem} causes the voltage to increase, and the voltage-gated Na channels activate and inactivate, followed by a delayed activation of the K channel. The whole system can be modeled using four differential equations given by Hodgkin and Huxley.

In the circuit of the Hodgkin-Huxley model shown Figure 2d, the relation between neuron membrane voltage V_{mem} and the influx of current $I_{in}(t)$ can be written as[4]:

$$I_{in}(t) = C_{mem}\frac{dV_{mem}}{dt} - \sum_k I_k(t), \quad (1)$$

where C_{mem} is the membrane capacitance and $\sum_k I_k(t)$ is the sum of the ionic currents passing through the membrane in biological terms. Hodgkin and Huxley further formulated the three ionic currents of K channel, Na channel, and the leakage as

$$\sum_k I_k(t) = g_K n^4(V_{mem} - E_K) + g_{Na} m^3 h(V_{mem} - E_{Na}) + g_L(V_{mem} - E_L), \quad (2)$$

where E_K, E_{Na}, and E_L are the reversal potentials and *n*, *m*, and *h* are ion channel gating variables that control the activation of the K channel, the activation of the Na channel, and the inactivation of the Na channel, respectively.

Supplementary Note 4. OECT HH neuron circuit analysis and SPICE simulations.

The conductance of the K-OECT has a sigmoidal behaviour dependent on V_{mem} based on its mobility μ_k, threshold voltage V_K , and time (delay) defined by R_{dk} and intrinsic capacitance C_{dk} analogous to the K activation variable n in HH model. For the Na-OECT, the conductance has a gaussian behaviour defined by two threshold voltages (V_{Na-m}, V_{Na-s}) and mobilities (μ_{Na-m}, μ_{Na-s}) corresponding to the multiply and singly charged species on either side of the gaussian similar to the activation (m) and inactivation (h) variables of Na in HH model. E_k and E_{Na} are constants and act as sodium and potassium batteries defining the lower and upper bounds of action potential; E_k also helps to modulate the threshold V_k of K-OECT. μ_k, V_{Na-m}, μ_{Na-m}, V_{Na-s} and μ_{Na-s} are functions of V_{mem}, while V_{mem} is a function of time defined by the charging of the

capacitance C_{mem}. Hence, the properties of the c-OECN can be described by these coupled state variables.

As shown in **Figure 2c**, the Hodgkin-Huxley neuron circuit based on BBL OECTs addresses the ionic channel (K and Na) conductance and their activation and inactivation, whereas the leakage channel is simply left to the device leakage in the circuit. The HH neuron model in Eq. 1 can be rewritten as follows:

$$I_{in}(t) = C_{mem}\frac{dV_{mem}}{dt} + i_K(t) - i_{Na}(t), \quad (3)$$

where $i_K(t)$ and $i_{Na}(t)$ are the ionic currents passing through the K channel and the Na channel respectively.

Given the K-OECT operates in a linear region when activated, the current of the K channel is therefore expressed as:

$$i_K(t) = \mu_K C_V \frac{Wd}{L}\left[\left(V_{mem}\left(1 - e^{\frac{t}{R_{dk}C_{dk}}}\right) - E_K - V_K\right)(V_{mem} - E_K) - \frac{(V_{mem}-E_K)^2}{2}\right], \quad (4)$$

where C_V is the volumetric capacitance of BBL polymer semiconductor, W, L, and d are the channel width, length, and thickness respectively, R_{dk} and C_{dk} provide the activation delay of the K-OECT. In the absence of C_{dk}, the gate capacitance of the K-OECT serves the same function.

The voltage to the gate of the Na-OECT is amplified with either inverting or non-inverting amplifier as discussed above and is a function of membrane voltage as $f(V_{mem}, t)$. The drain of the Na-OECT is applied with E_{Na} while the source of the Na-OECT is connected to the node of V_{mem}. Two thresholds $V_{Na\text{-}m}$ and $V_{Na\text{-}s}$ are employed to simulate the activation and the inactivation of the Na channel, respectively. The current of the Na channel is then written as a combination of two equations of OECTs operated in saturation region as:

$$i_{Na}(t) = \mu_{Na-m} C_V \frac{Wd}{L}[f(V_{mem}, t) - V_{mem} - V_{Na-m}]^2, \ (V_{NA\text{-}m} < f(V_{mem}) < (V_{NA\text{-}m} + V_{NA\text{-}s})/2) \quad (5)$$

$$i_{Na}(t) = \mu_{Na-s} C_V \frac{Wd}{L}[f(V_{mem}, t) - E_{Na} - V_{Na-s}]^2, \ ((V_{NA\text{-}m} + V_{NA\text{-}s})/2 < f(V_{mem}) < V_{NA\text{-}s}). \quad (6)$$

To further simulate the behaviors of c-OECNs, SPICE models based on equivalent circuits have been developed for both K-OECT and Na-OECT. The K-OECT SPICE model is a further update on the previously reported[5], with larger channel (W/L= 400/6 μm). The simulated transfer characteristics of K-OECT is presented in **Supplementary Figure 33a** along with the

measured data. For the Na-OECT, a new equivalent circuit has been developed with a pair of PMOS (Ms) and NMOS (Mm) transistors, shown in **Supplementary Figure 32**. The simulated transfer characteristics of Na-OECT is shown in **Supplementary Figure 33b**.

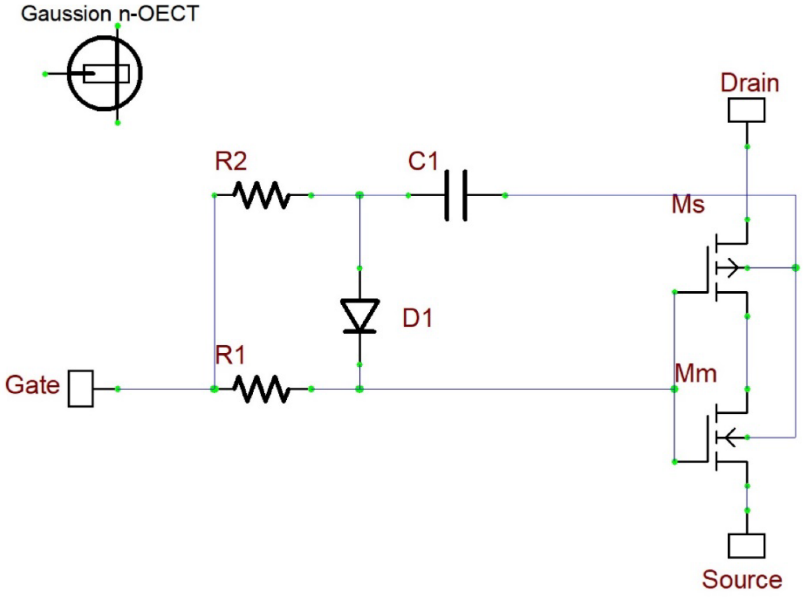

Supplementary Figure 32. Left upper: Symbol of the SPICE model for the BBL n-type OECTs with antiambipolar/Gaussian characteristics. The equivalent circuit consists of two resistors (R2=800 kΩ, R1=850 kΩ), one capacitor (C1=90 nF), one diode (D1), one PMOS (Ms), and one NMOS (Mm).

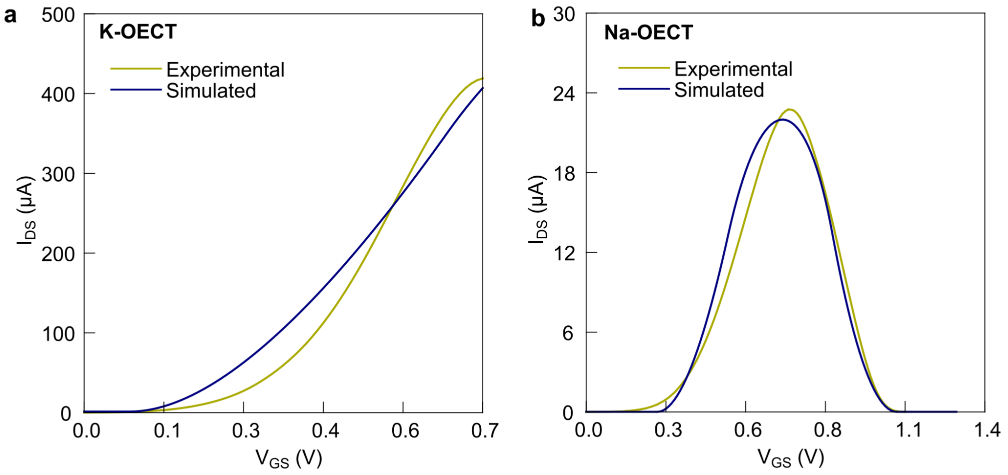

Supplementary Figure 33. Comparison of simulated and measured transfer characteristics of Na and K-OECTs.

With the SPICE models built, we simulate various spiking features of c-OECN, and **Supplementary Figure 34** presents a typical tonic spiking circuit. The simulated c-OECN spiking features are shown in **Supplementary Figure 35**.

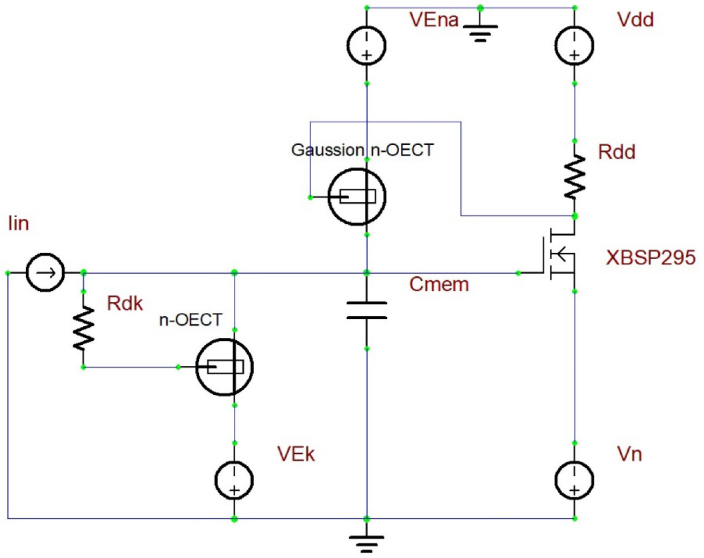

Supplementary Figure 34. A typical tonic spiking c-OECN with SPICE models.

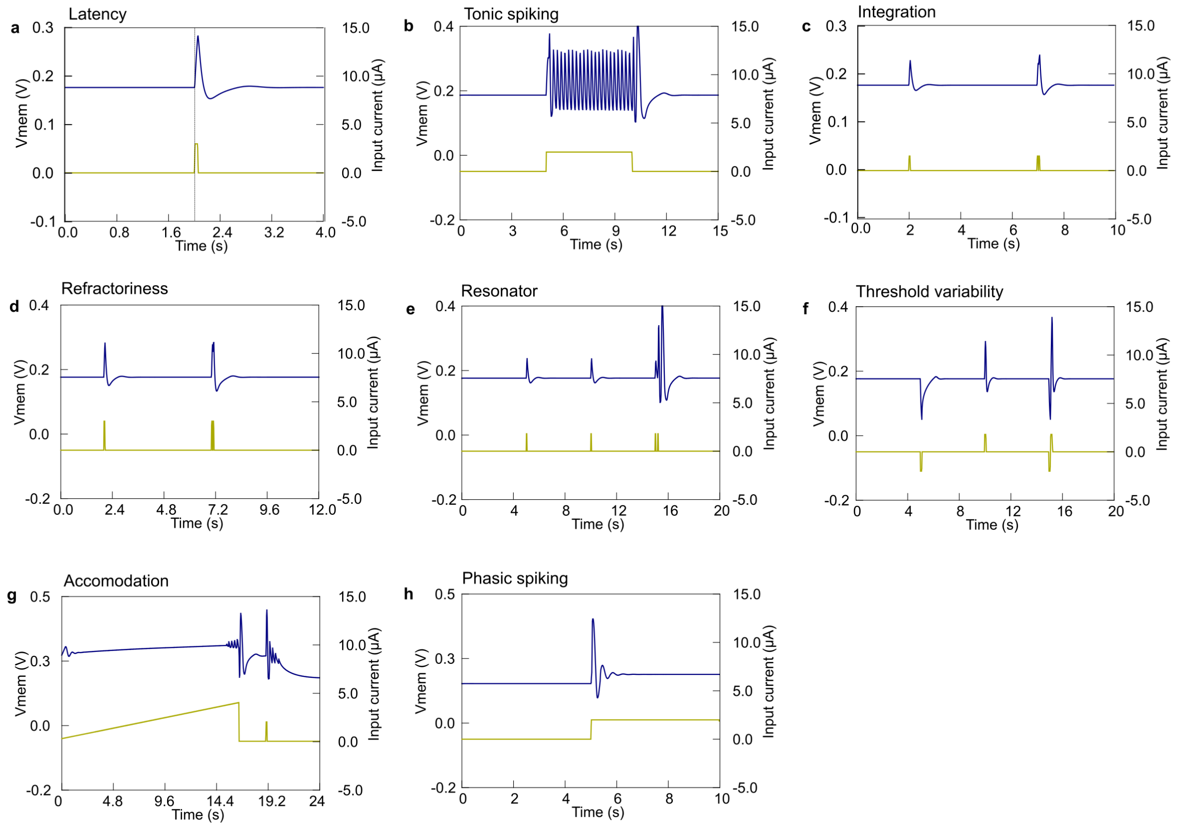

Supplementary Figure 35. Neural features based on SPICE simulations with K-OECT and Na-OECT models.

For comparison, we also investigate the possibility of using an NMOS transistor instead of K-OECT to represent the K channel, where one extra capacitor C_{dk} is needed to adjust the delay of the activation of the K channel **(Supplementary Figure 36)**. It suggests that K-OECT can be replaced by other n-type enhacement-mode transistors (for example, NMOS, TFTs, etc.), however, the Na-OECT relies on the unique feature of the antiambipolar BBL OECTs. The experimental and simulated neural features of this circuit are shown in **Supplementary Figures 37 and 38**.

The gaussian curve of the Na-OECT shifts towards higher voltages on increasing its V_{DS}. In our typical circuit with two OECTs and the NMOS inverter, E_{Na} of 0.5 V is chosen because it is the highest safe operating voltage to have the gaussian transfer curve turn off at a voltage of 1.2 V (to avoid water electrolysis reactions). Ek is chosen to adjust the threshold of K-OECT to discharge the Na-OECT completely. Hence, it will vary slightly from around -0.05 to +0.1V depending on the current level and threshold voltage of the K-OECT employed. We adjusted the voltage range by gradually increasing the voltage to find the optimum point of spiking. The value of Ek also determines the resting potential of the neuron. Hence to bring the baseline of the neuron to lower values, it is sufficient to use a K-OECT with a higher threshold voltage and then adjust the Ek to more negative values.

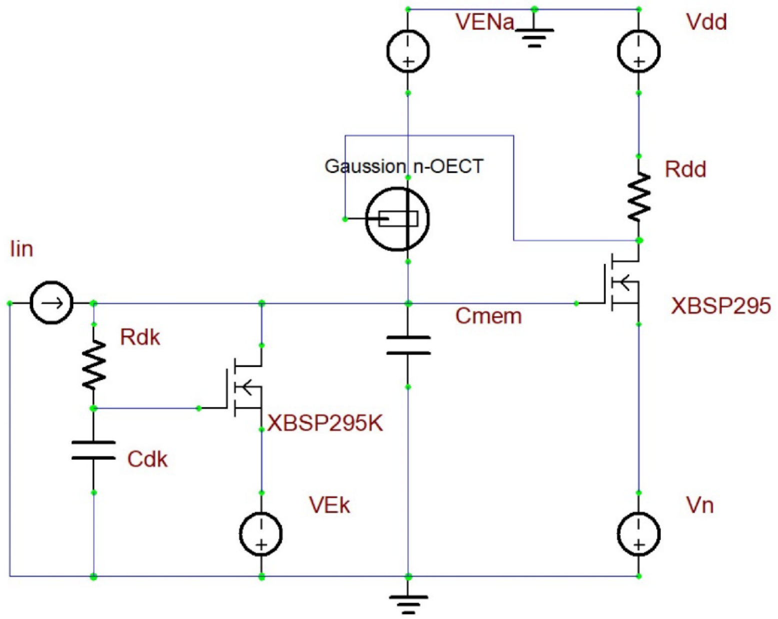

Supplementary Figure 36. A similar c-OECN as Supplementary Figure 25, with an NMOS transistor to replace the K-OECT.

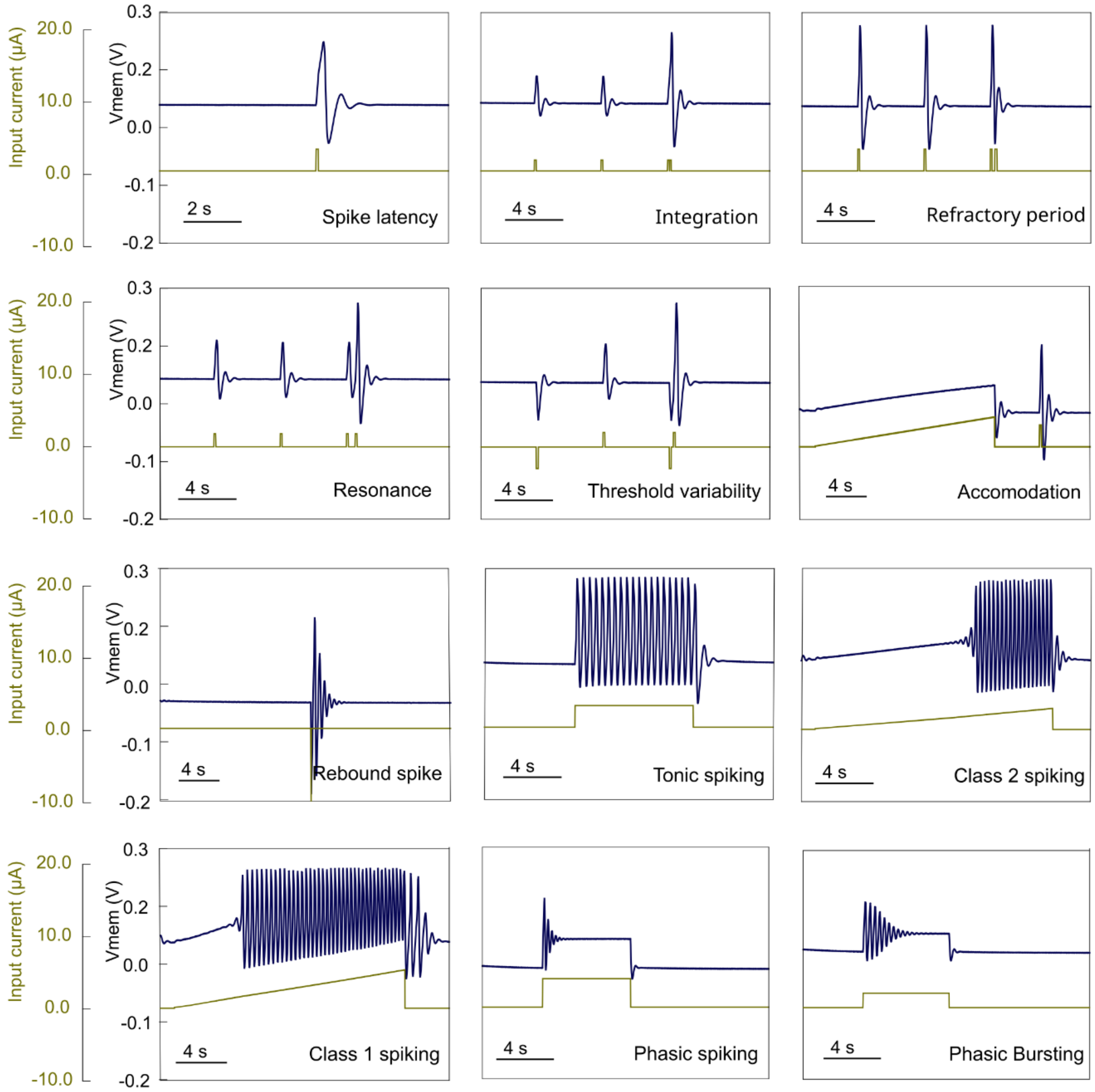

Supplementary Figure 37. Features of the neuron using an NMOS transistor along with capacitor C_{dk} instead of OECT for the K-channel.

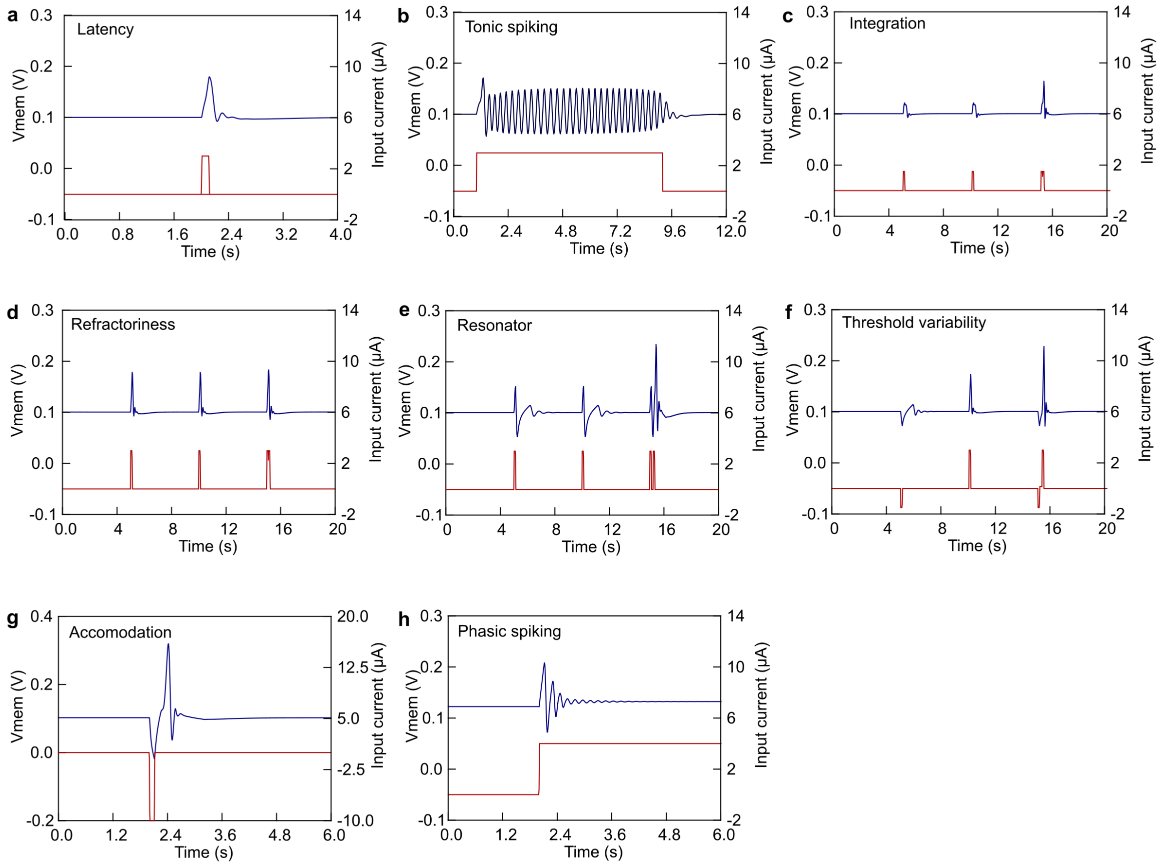

Supplementary Figure 38. Simulated neuron features using an NMOS transistor and capacitor C_{dk} instead of OECT for the K-channel.

Supplementary Note 5. Power consumption and energy efficiency estimation and downscaling.

As shown in **Supplementary Figure 34**, the artificial neuron circuit uses four voltage sources and one current source. Therefore, the total power consumption P_{tot} is calculated as the sum of all power sources consumed, as:

$$P_{tot} = P_{in} + P_{Na} + P_K + P_{INV} \quad (7a)$$

$$P_{in} = V_{mem} \times I_{in} \quad (7b)$$

$$P_{Na} = (E_{Na} - V_{mem}) \times I_{Na} \quad (7c)$$

$$P_K = (V_{mem} - E_K) \times I_K \quad (7d)$$

$$P_{INV} = (V_{DD} - Vn) \times I_{RDD} \quad (7e)$$

where P_{in} represents the input current source, P_{Na} and P_K are the Na and K channels, and P_{INV} is the inverter amplifier.

Supplementary Figure 39a shows the sample action potential of a slow neuron with a period of 350 ms and the corresponding Na and K currents. For the same period, the power consumption of each source is shown (**Supplementary Figure 39b**), where the main contribution is from P_{INV} (max 49.6 μW). Although we have reported complementary OECT-based inverter amplifiers consuming less than 1 μW of power[6], here we used NMOS amplifiers with a resistor load because of the desired output voltage and stability (>60 min continuously spiking). The second large contribution is from the K channel, which consumes 5.7 μW. The Na channel and the current input consume 2.5 μW and 0.98 μW in the peak, respectively **(Supplementary Figure 39c).** The maximum total power consumption reaches 55.7 μW and the summed power of the Na channel, K channel, and input current (excluding the inverter amplifier) is 9 μW (**Supplementary Figure 39d**).

The energy consumed by this slow neural spiking (350 ms time period) obtained by integrating the power over time is 1100 nJ/spike (**Supplementary Figure 39d**) excluding the inverter amplifier, and 4900 nJ/spike including the amplifier. Since the same circuit has been used to realize neurons spiking at 80 Hz (12.5 ms time period), the energy consumption of such fast spiking is about 39 and 175 nJ/spike without and with the inverter amplifier, respectively.

So, if we consider biorealistic frequencies and spike widths which are necessary for efficient interaction with other biological neurons and the real world, the energy consumption of the c-OECNs will be lower compared to conductance-based Si neurons with further optimization on the inverter amplifier. The energy consumption of the c-OECNs is similar to 2D chalcogenide heterojunction-based devices (200 nJ)[7]. The main component of power consumption is the NMOS-based inverter which can be reduced using alternative low-power OECT-based inverters (<1 μW)[5]. However, such complementary OECT inverters can only be implemented once they are stable enough, and the V_P of gaussian can be modified to be in their switching range. To investigate the energy efficiency of the c-OECNs, we run simulations of power consumption dependence on the OECT scaling (excluding the inverter). Two downscaling cases are compared:

a) W/L ratio is kept constant (L = 6, 3, 1.5 μm)

b) L is kept constant, while W is varied (40, 20, 10 μm) for the Na-OECT.

Both K-OECT and Na-OECT channels are downscaled to the same ratio, along with the membrane capacitor (C_{mem} = 1 µF, 500 nF, 250 nF, respectively). The inverter amplifier and the voltage source are kept constant for the simulation. **Supplementary Figure 40a** shows that the c-OECN's peak power consumption (excluding P_{INV}) increases slightly when the OECTs are downscaled keeping the W/L ratio constant, while it decreases significantly when L is kept constant, and W is varied. Therefore, with higher resistance scaled devices, the OECN would indeed consume less power. However, the downscaling of W/L shows a significantly higher spiking frequency (**Supplementary Figure 40b**) when L is reduced to 1.5 µm. Thus, the overall energy consumption per spike (excluding the inverter's contribution) is reduced significantly in both cases (**Supplementary Figure 40c**). This is in line with our previous work, where we demonstrated that switching speed increases linearly with a reduction in parasitic capacitances on scaling[5]. It should be noted that in the simulation, we did not scale the voltage sources or the input current. In principle, scaling the dimensions will also enable scaling down these voltages and input currents resulting in further reduction in power consumption according to Dennard power scaling[8], a well-proven concept in silicon transistors. For example, in OECT-based inverters with reduced dimensions, low voltage inputs are sufficient to enable significant gain, resulting in low power consumption[6,9]. Furthermore, downscaling can be combined with suitable material engineering (for example, diluting the semiconductor with an insulating polymer) to reduce the conductivity of the channel and hence the current through channels which determine the power consumption.The energy consumption of the inverter can be scaled similarly if complementary OECT based inverters are implemented in the circuit.

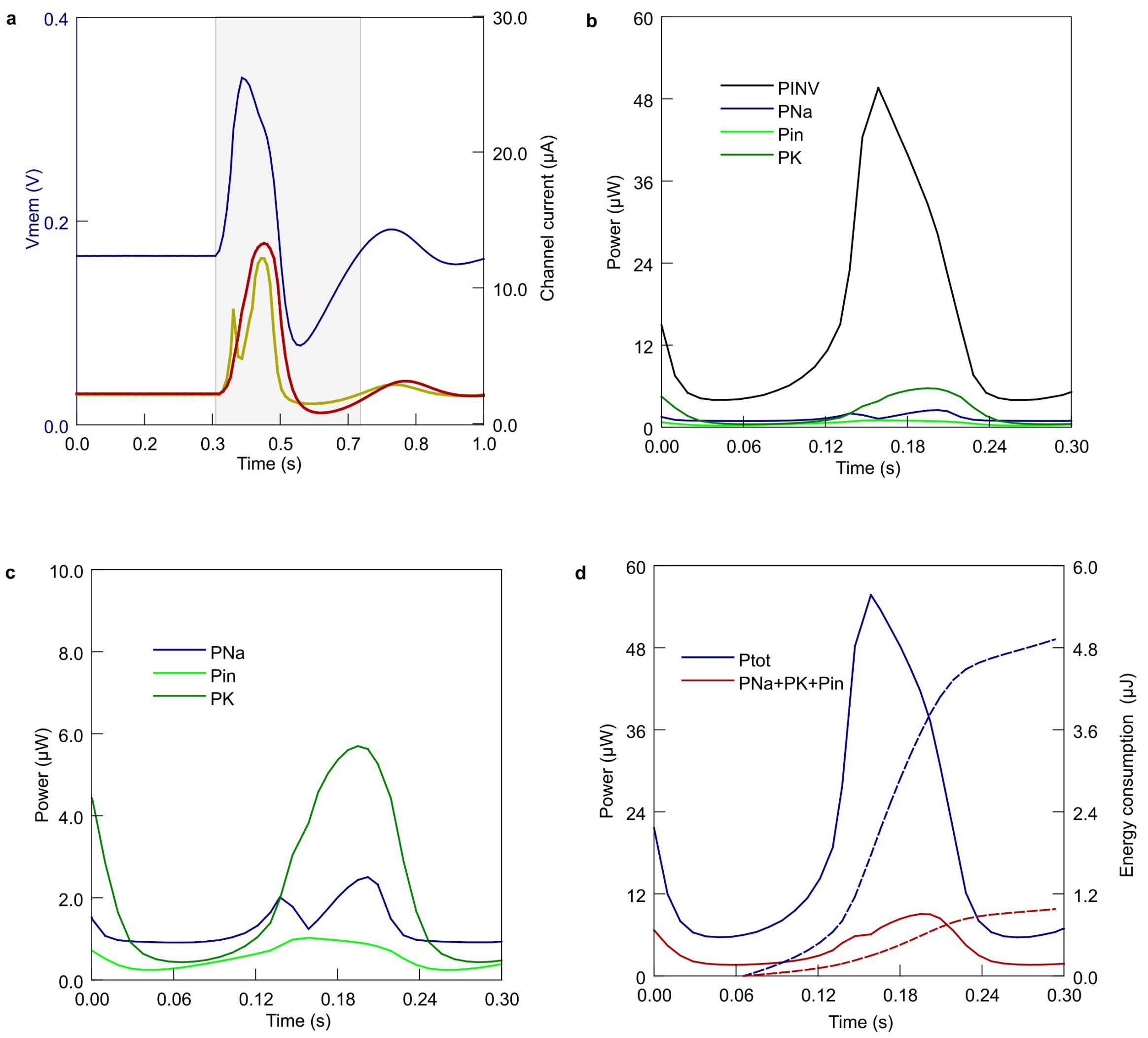

Supplementary Figure 39. (a) Sample action potential of a slow neural spike (period of 350ms) showing the Na^+ and K^+ currents and (b-d) the corresponding power and energy consumption.

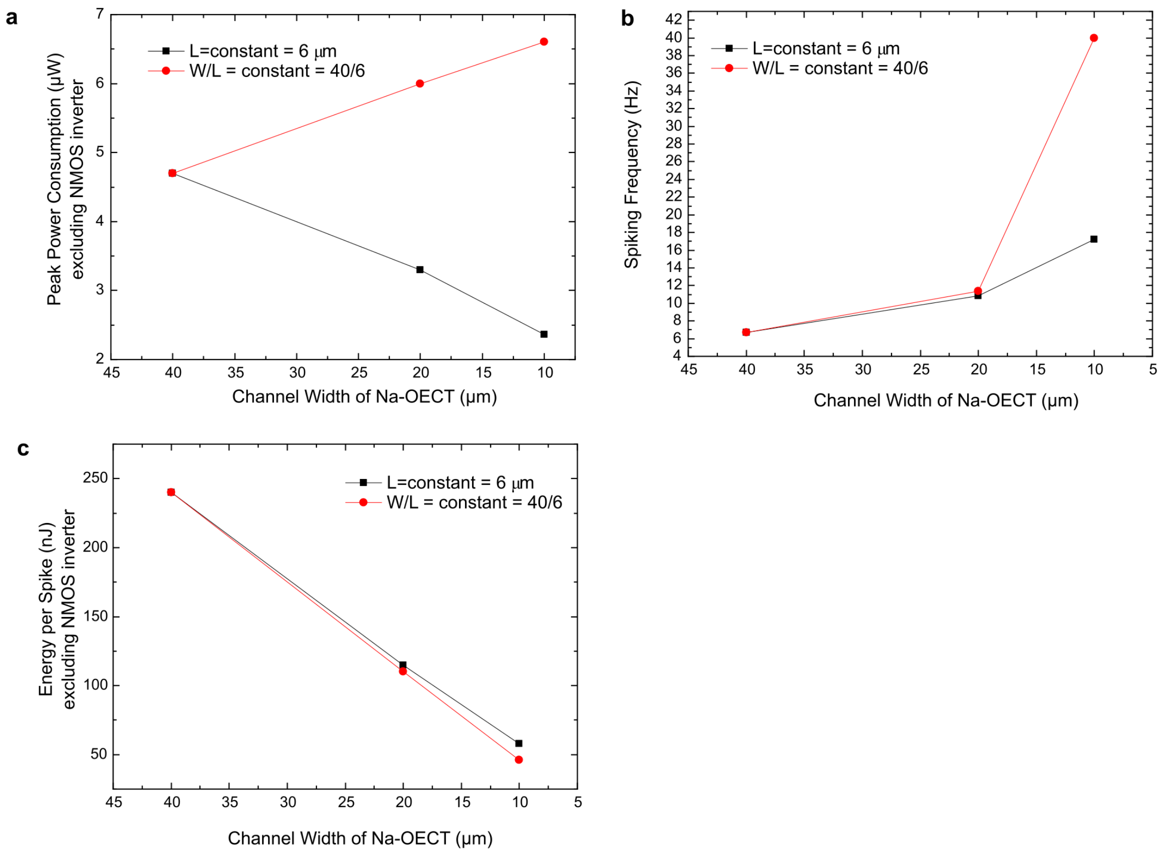

Supplementary Figure 40. (a-c) The power frequency and energy/spike variation (excluding the contribution from the NMOS-based inverting amplifier) when scaling the channel width, keeping L as constant and W/L as constant.

Supplementary Note 6. Features of neuron.

It is observed that there is a delay between the applied input pulse and the generated spike in the c-OECN (**Figure 3d**). This latency encodes the input pulse strength because this delay will be proportional to the input amplitude. The OECN can demonstrate the integration of the input signals, ie when two inputs producing subthreshold spikes occur in a short interval of time, it is integrated to generate a spike and enable coincidence detection (**Figure 3e**). However, if a second input occurs during the hyperpolarisation phase of a previous action potential, there is no new spike generation, thus exhibiting refractoriness (**Figure 3f**). Due to the presence of subthreshold oscillations associated with action potential, the c-OECN exhibits resonance or frequency preference, ie when two subthreshold inputs occur at a frequency in phase with the oscillations, a spike is generated (**Figure 3g**). This enables frequency modulated interactions

with the neuron. As opposed to the leaky integrate and fire-based OECN which exhibits a fixed threshold for spike generation, the c-OECN has a variable threshold that depends on the neuron's prior activity. As shown in **Figure 3h**, a signal which generates subthreshold output can create a spike if the threshold is lowered by a preceding inhibitory input. Rebound spike, where the c-OECN spikes in response to an inhibitory input (**Figure 3i**), and accommodation where a slowly varying input does not invoke a spike while a smaller but sharper input (**Figure 3j**) can incite a spike can also be observed.

The c-OECN can emulate 3 classes of neurons by varying the threshold of the K-OECT (Figure 3k-m). For an E_k= -0.05 V, class 1 spiking is observed where the spiking frequency varies with the strength of the input. These neurons can encode the strength of input in frequency of spiking. Changing the E_k to more negative values of -0.065V changes the behavior to class 2 spiking where the c-OECN spikes at high amplitude inputs without much frequency modulation. An even larger negative E_k causes Phasic bursting and spiking which is commonly referred to as class 3 spiking where spikes or bursts are generated at the onset of the input helping the encode the beginning of stimulation. Bursting helps reduce synaptic failure, transmit input saliency, and enable selective communication between neurons. The neural circuit used to emulate these features is shown in **Supplementary Figures 44 and 45**

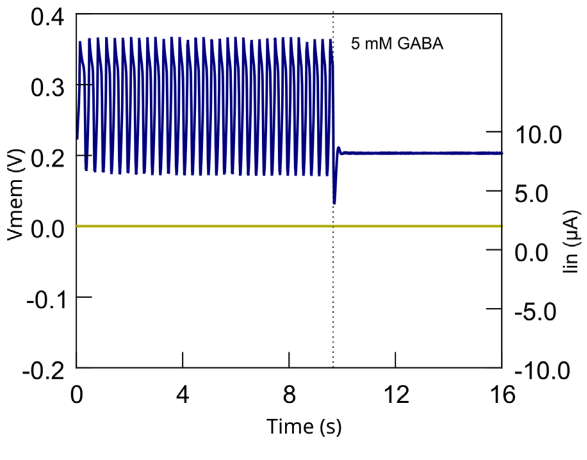

Supplementary Figure 41. The inhibition of spiking using GABA.

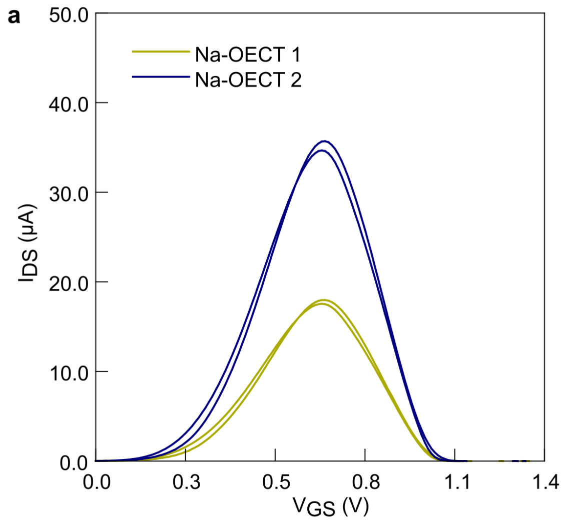

Supplementary Figure 42. Transfer characteristics of the Na-OECTs and K-OECTs used in the circuit to obtain various neural features are in Supplementary Table 1.

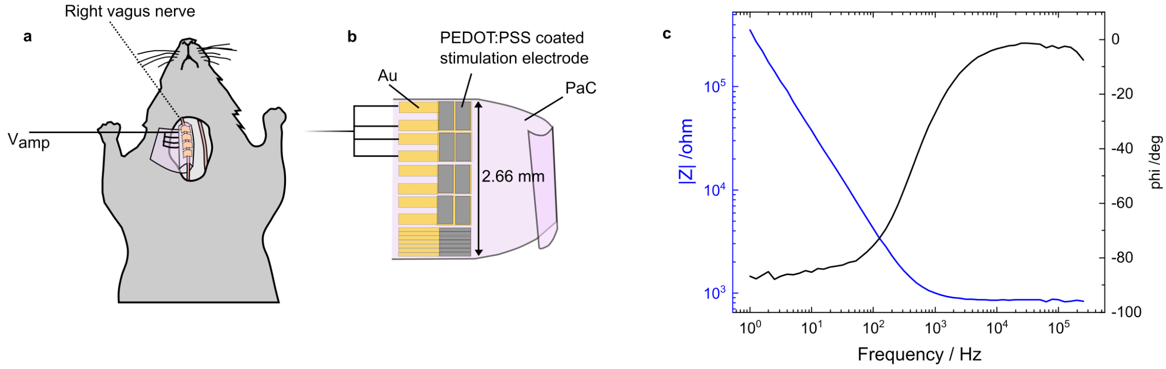

Supplementary Figure 43. (a) Interfacing the cuff electrodes with the right vagus nerve. (b) The structure of the cuff electrode. (c) Impedance measurements of the electrode.

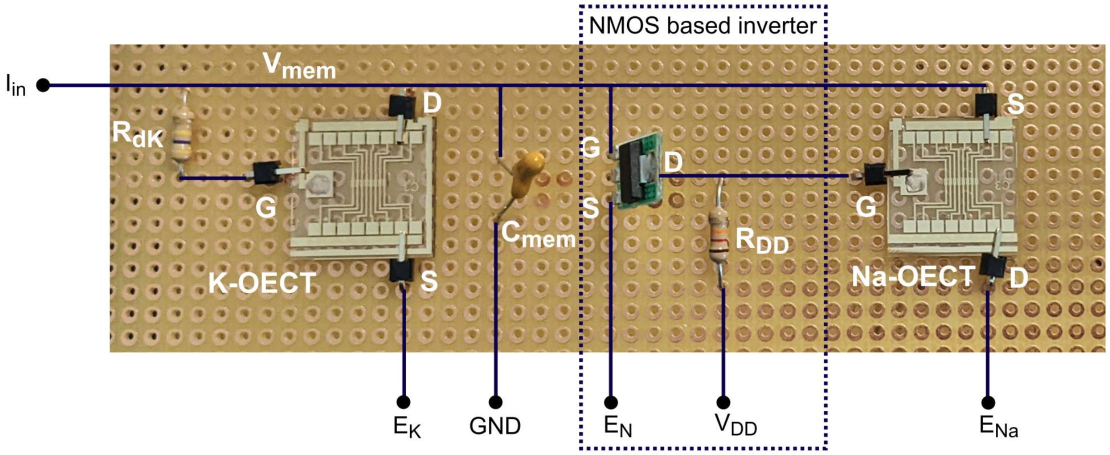

Supplementary Figure 44. Photograph of the c-OECN with 2 OECTs and NMOS based inverter. (C_{mem} is optional). The connections depicted by black lines are below the circuit board.

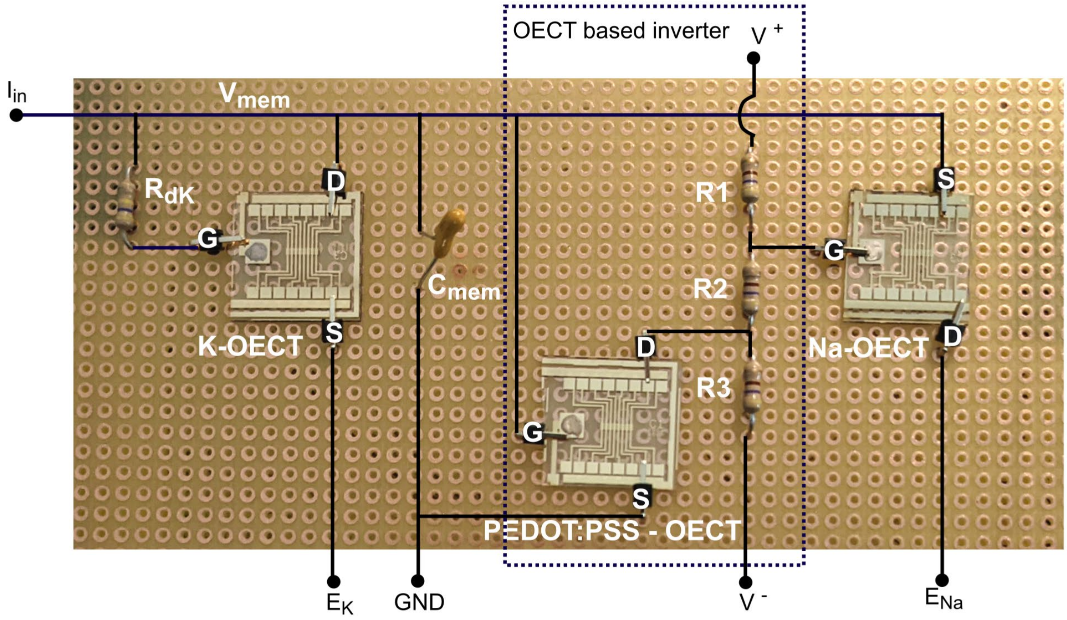

Supplementary Figure 45. Photograph of the c-OECN with 3 OECTs (C_{mem} is optional). The connections depicted by black lines are below the circuit board.

Supplementary Table 1. Parameters used for different configurations of c-OECN.

	Feature	Na-OECT	K-OECT	C_{mem} (μF)	R_{dk} (kΩ)	E_k (V)	E_{Na} (V)	V_{DD} (V)	E_N (V)	R_{DD} (kΩ)
1	Tonic spiking	Na-OECT 1 in Supplementary Figure 42	K-OECT 1 in Supplementary Figure 42	1	470	-0.05	0.5	1.25	-0.66	12
2	Latency									
3	Subthreshold oscillations									
4	Integration									
5	Refractoriness									
6	Resonance									
7	Variable Threshold									
8	Rebound spike									
9	Accomodation									
10	Phasic Bursting					-0.07				
11	Phasic spiking					-0.072				
12	Class 1 spiking					-0.05				
13	Class 2 spiking					-0.065				
15	Calcium control					-0.05				
16	GABA control	Na-OECT 2 in Supplementary Figure 42	K-OECT 2 in Supplementary Figure 42			-0.012			-0.65	
14	Stochastic spiking					0.025				
17	Fast neuron (45Hz)			-	68	0.01				
18	Fast neuron (80Hz)			-	27	0.02				

Supplementary Table 2. Comparison of artificial neurons based on various technologies.

	Circuit elements (excluding power sources)	Features	Switching speed of channel	Ion based modu-lation	Spike voltage swing (mV)	Frequency	Power / Energy consumption of a spike
Silicon CMOS (biorealistic conductance-based models)[10–19]	14 to 30+ [13]transistors + passive components	~20	< 1 ns	No	120[19] - 3000[13]	100 Hz to MHz	8 – 60[11] µW / < 100 fJ to 1 nJ
Mott-Memristors[20,21]	2 memristors 3 resistors 3 capacitors	~20	< 1ns	No	400 - 1200	10 to 50 kHz	2 mW / ~ > 10 nJ
2D material-based Gaussian heterojunction[7]	3 to >10 transistors 2 to 3 resistors 1 to 3 capacitors	8 (simulat ed)	-	No	~ 400	< 1Hz	~ 1.25 µW/ 250 nJ
OFET (LIF)[22]	5 transistors 2 capacitors	3	-	No	~ 3000	1 to 5 Hz	40 µW / ~20 µJ
Complementary OECT (LIF)[5]	5 transistors 2 capacitors (optional)	3	200 - 400 ms	Yes	~ 500	0.25 Hz	15 µW / ~15 µJ
Antiambipolar-OECT (this work)	**3 transistors 2 resistors 1 capacitor (optional)**	15	**0.5 - 1.5 ms**	**Yes**	**100 - 200**	**100 Hz**	58.6 µW / 175 nJ
Biological neuron		~20 known	0.5 to 10 ms	Yes	~ 120	0.5 Hz to 500 Hz	100 pJ[23]

References :

1. Maheshwari, S., Patwardhan, S., Schatz, G. C., Renaud, N. & Grozema, F. C. The effect of the magnitude and direction of the dipoles of organic cations on the electronic structure of hybrid halide perovskites. *Phys. Chem. Chem. Phys.* **21**, 16564–16572 (2019).

2. Tian, Z. *et al.* High-Capacity NH4+ Charge Storage in Covalent Organic Frameworks. *J. Am. Chem. Soc.* **143**, 19178–19186 (2021).

3. Medhi, I. & Iyer, P. K. An engineered organic electrochemical transistor (OECT) platform with a highly ammonia-sensitive mesoporous membrane. *Sens. Diagn.* (2022) doi:10.1039/D2SD00099G.
4. Gerstner, W., Kistler, W. M., Naud, R. & Paninski, L. *Neuronal Dynamics: From Single Neurons to Networks and Models of Cognition*. (Cambridge University Press, 2014).
5. Harikesh, P. C. *et al.* Organic electrochemical neurons and synapses with ion mediated spiking. *Nat. Commun.* **13**, 901 (2022).
6. Wu, H.-Y. *et al.* Influence of Molecular Weight on the Organic Electrochemical Transistor Performance of Ladder-Type Conjugated Polymers. *Adv. Mater.* **34**, 2106235 (2022).
7. Beck, M. E. *et al.* Spiking neurons from tunable Gaussian heterojunction transistors. *Nat. Commun.* **11**, 1565 (2020).
8. Dennard, R. H. *et al.* Design of ion-implanted MOSFET's with very small physical dimensions. *IEEE J. Solid-State Circuits* **9**, 256–268 (1974).
9. Yang, C.-Y. *et al.* Low-Power/High-Gain Flexible Complementary Circuits Based on Printed Organic Electrochemical Transistors. *Adv. Electron. Mater.* **8**, 2100907 (2022).
10. Indiveri, G. *et al.* Neuromorphic silicon neuron circuits. *Front. Neurosci.* **5**, 73 (2011).
11. Mahowald, M. & Douglas, R. A silicon neuron. *Nature* **354**, 515–518 (1991).
12. Livi, P. & Indiveri, G. A current-mode conductance-based silicon neuron for address-event neuromorphic systems. in *2009 IEEE International Symposium on Circuits and Systems* 2898–2901 (2009). doi:10.1109/ISCAS.2009.5118408.
13. Wijekoon, J. H. B. & Dudek, P. Compact silicon neuron circuit with spiking and bursting behaviour. *Neural Netw.* **21**, 524–534 (2008).
14. Folowosele, F., Etienne-Cummings, R. & Hamilton, T. J. A CMOS switched capacitor implementation of the Mihalas-Niebur neuron. in *2009 IEEE Biomedical Circuits and Systems Conference* 105–108 (2009). doi:10.1109/BIOCAS.2009.5372072.

15. Patel, G. N. & DeWeerth, S. P. Analogue VLSI Morris-Lecar neuron. *Electron. Lett.* **33**, 997–998 (1997).

16. van Schaik, A., Jin, C., McEwan, A. & Hamilton, T. J. A log-domain implementation of the Izhikevich neuron model. in *Proceedings of 2010 IEEE International Symposium on Circuits and Systems* 4253–4256 (2010). doi:10.1109/ISCAS.2010.5537564.

17. Akbari, M., Hussein, S. M., Chou, T.-I. & Tang, K.-T. A 0.3-V Conductance-Based Silicon Neuron in 0.18 μm CMOS Process. *IEEE Trans. Circuits Syst. II Express Briefs* **68**, 3209–3213 (2021).

18. Ronchini, M., Zamani, M., Farkhani, H. & Moradi, F. Tunable Voltage-Mode Subthreshold CMOS Neuron. in *2020 IEEE Computer Society Annual Symposium on VLSI (ISVLSI)* 252–257 (2020). doi:10.1109/ISVLSI49217.2020.00053.

19. Sourikopoulos, I. *et al.* A 4-fJ/Spike Artificial Neuron in 65 nm CMOS Technology. *Front. Neurosci.* **11**, (2017).

20. Pickett, M. D., Medeiros-Ribeiro, G. & Williams, R. S. A scalable neuristor built with Mott memristors. *Nat. Mater.* **12**, 114–117 (2013).

21. Yi, W. *et al.* Biological plausibility and stochasticity in scalable VO 2 active memristor neurons. *Nat. Commun.* **9**, 4661 (2018).

22. Hosseini, M. J. M. *et al.* Organic electronics Axon-Hillock neuromorphic circuit: towards biologically compatible, and physically flexible, integrate-and-fire spiking neural networks. *J. Phys. Appl. Phys.* **54**, 104004 (2020).

23. Lee, Y., Park, H.-L., Kim, Y. & Lee, T.-W. Organic electronic synapses with low energy consumption. *Joule* **5**, 794–810 (2021).
